# More than just protein building blocks: how amino acids and related metabolic pathways fuel macrophage polarization

**DOI:** 10.1111/febs.15715

**Published:** 2021-02-22

**Authors:** Markus Kieler, Melanie Hofmann, Gernot Schabbauer

**Affiliations:** ^1^ Institute for Vascular Biology Centre for Physiology and Pharmacology Medical University Vienna Vienna Austria; ^2^ Christian Doppler Laboratory for Arginine Metabolism in Rheumatoid Arthritis and Multiple Sclerosis Vienna Austria

**Keywords:** arginase/iNOS, glutamine, immunometabolism, macrophage polarization, nitric oxide, oxidative phosphorylation, polyamines, serine, TCA cycle, α‐ketoglutarate

## Abstract

Macrophages represent the first line of defence in innate immune responses and additionally serve important functions for the regulation of host inflammation and tissue homeostasis. The M1/M2 model describes the two extremes of macrophage polarization states, which can be induced by multiple stimuli, most notably by LPS/IFN‐γ and IL‐4/IL‐13. Historically, the expression of two genes encoding for enzymes, which use the same amino acid as their substrate, iNOS and ARG1, has been used to define classically activated M1 (iNOS) and alternatively activated M2 (ARG1) macrophages. This ‘arginine dichotomy’ has recently become a matter of debate; however, in parallel with the emerging field of immunometabolism there is accumulating evidence that these two enzymes and their related metabolites are fundamentally involved in the intrinsic regulation of macrophage polarization and function. The aim of this review is to highlight recent advances in macrophage biology and immunometabolism with a specific focus on amino acid metabolism and their related metabolic pathways: iNOS/ARG1 (arginine), TCA cycle and OXPHOS (glutamine) as well as the one‐carbon metabolism (serine, glycine).

## Graphical abstract

Macrophages are key players in innate immunity and react to a great variety of pro‐ and anti‐inflammatory stimuli, which result in a continuum of polarization states. The M1/M2 model describes the two extremes of this spectrum. Emerging evidence points towards a key role of metabolism in immunobiology and highlights the importance of specific amino acids and their related metabolic pathways in macrophage polarization.

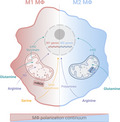

**Abbreviations**
ACO2: aconitase 2
ACOD1: aconitate decarboxylase 1
ARG1: arginase 1
ATP: adenosine triphosphate
CD206: mannose receptor
DLD: dihydrolipoamide dehydrogenase
eIF5A: eukaryotic translation initiation factor 5A
ETC: electron transport chain
FAO: fatty acid oxidation
GABA: gamma aminobutyric acid
GLS: glutaminase
GS: glutamine synthetase
H3K27: histone H3 lysine‐27
HIF‐1α: hypoxia‐inducible factor 1 alpha
IFN‐γ: interferon gamma
IL‐1β: interleukin‐1 beta
iNOS: inducible nitric oxide synthase
Irg1: immunoresponsive gene 1
IRS: insulin receptor substrate
JAK: janus kinase
JMJD3: jumonji domain‐containing 3
LPS: lipopolysaccharide
MGL2: macrophage galactose N‐acetyl‐galactosamine‐specific lectin 2
mTOR: mechanistic target of rapamycin
NRF2: nuclear factor erythroid‐2‐related factor 2
Nuclear factor kappa‐light‐chain‐enhancer of activated B cells: NF‐κB
OGDC: oxoglutarate dehydrogenase complex
OXPHOS: oxidative phosphorylation
PDHC: pyruvate dehydrogenase complex
PHD: prolyl hydroxylases
PHGDH: phosphoglycerate dehydrogenase
PPAR‐γ: peroxisome proliferator‐activated receptor‐gamma
RELMα: resistin‐like molecule‐alpha
ROS: reactive oxygen species
SAM: S‐adenosyl methionine
SDH: succinate dehydrogenase
SSP: serine synthesis pathway
STAT6: signal transducer and activator of transcription 6
TCA cycle: tricarboxylic acid cycle
Th_1_/Th_2_
: type I/type II helper T cell
TLR: Toll‐like receptor
UDP‐GlcNac: uridine diphosphate N‐acetylglucosamine
α‐KG: alpha‐ketoglutarate

## Introduction

Nearly one century ago, Otto F. Warburg realized that slices of tumour tissues convert glucose to lactate much faster than healthy tissues [1]. Interestingly, this rapid lactate production even happens in the presence of sufficient oxygen, which would allow respiration via oxidative phosphorylation (OXPHOS) in mitochondria. This observation has later been termed the ‘Warburg effect’. Warburg also found a similar metabolic phenotype in leucocytes but falsely attributed it to an artefact of preparation [2]. However, earlier studies had already demonstrated that neutrophils and macrophages are similarly dependent on the process of aerobic glycolysis for the production of adenosine triphosphate (ATP) [3, 4, 5, 6]. The evidence for increased glucose uptake and a switch to aerobic glycolysis in lymphocytes upon stimulation with mitogens was underpinned by a series of studies starting in the 1960s [7, 8, 9, 10, 11, 12]. Increased lactate production of cells, which are dividing, was observed at the same time and led to the notion that aerobic glycolysis is indeed a hallmark of cellular proliferation [13, 14, 15]. As more and more efficient, even cell type‐specific, gene‐modifying technologies were introduced, the field of immunometabolism gained new momentum. One of the first studies, which marked the beginning of the new era, demonstrated that hypoxia‐inducible factor 1 alpha (HIF‐1α) is essential for myeloid cell‐mediated inflammation by regulating ATP production [16]. In particular, in the last 10 years a rapidly growing number of studies have elucidated that metabolic processes such as aerobic glycolysis and respiration serve a much broader purpose that goes beyond proliferation and demonstrated that metabolic pathways distinctly shape the immune response.

While glucose metabolism has long been the focus and still is one of the major research areas of how metabolism shapes the immune response, immunologists now increasingly turn their attention to the impact of amino acid metabolism on immune cells, especially on macrophage function. Before understanding the mechanisms, it was a matter of debate, which type of cells contribute to the excess nitrate production in mammals [17]. In 1985, macrophages have been identified as a major source of this metabolite in the urine of lipopolysaccharide (LPS)‐treated mice [18]. Subsequent studies could show that a metabolic product of arginine mediates the cytotoxic and bacteriostatic effects of LPS‐activated macrophages [19, 20]. Over the last decade, it has become clear that additionally to its role as a key defence element, arginine‐derived NO is essential for orchestrating the metabolic adaptation during macrophage activation and thus ultimately controls the cell’s response to inflammatory stimuli. Although receiving less attention, studies reporting alternative arginine conversion via arginase 1 (ARG1) were published in parallel and at first mainly linked ARG1 activity to suppression of cytotoxic T cell function in mixed macrophage lymphocyte cultures [21]. Soon later in the 1990s, it was discovered that cytokines such as IL‐4, which inhibit NO production, upregulate ARG1 activity in macrophages [22, 23, 24]. The term classical (M1) and alternative (M2) macrophage activation stems from exactly these alternative metabolic states reflected by the nitric oxide synthase (iNOS)/ARG1 balance and furthermore correlates with type I T helper/type II T helper (Th_1_/Th_2_) cell phenotypes [25]. In accordance with the essential role of iNOS‐derived NO for the metabolic adaptation required for M1 macrophage activation, recent evidence suggests that also the alternative metabolic flux via ARG1 plays a fundamental role for M2 macrophages to acquire their phenotype and fulfil their effector functions. In this regard, it is important to note that iNOS and ARG1 are not exclusively expressed by either pro‐ or anti‐inflammatory myeloid subsets. In fact, due to the high plasticity of macrophages several studies reported the presence of myeloid cells that are positive for both enzymes in distinct pathological settings including *Mycobacterium tuberculosis (M. tuberculosis)* infection, the tumour immune infiltrate and a mouse model for multiple sclerosis [26, 27, 28]. Additionally, there is accumulating evidence that ARG1 expression might also be induced by pathways that are usually linked to an inflammatory innate immune response [27, 29, 30, 31, 32, 33], which further challenges this ‘arginine dichotomy’.

Besides the iNOS/ARG1 axis, the tricarboxylic acid (TCA) cycle has emerged as a central immunometabolic hub for macrophage activation. The observation that macrophages in response to LPS/IFN‐γ suppress OXPHOS and induce two functionally important disruptions in the TCA cycle, which lead to the accumulation of succinate and itaconate, stands in stark contrast to a fully functional TCA cycle and the high OXPHOS activity in IL‐4‐stimulated macrophages [34, 35, 36, 37]. Hence, both of these two TCA cycle modes play fundamental roles by regulating the activation state and functional properties of macrophages. Additionally, the amino acid glutamine has been shown to be crucial for both macrophage phenotypes by feeding into the TCA cycle via glutamate and alpha‐ketoglutarate (α‐KG). This is, however, triggered by different events during M1 and M2 activation, which have specific consequences in the context of the respective polarization state [38].

Additionally to glutamine, the involvement of amino acids in redox homeostasis such as glycine and serine has sparked new interests in the field of macrophage biology. Recently, the serine synthesis pathway, which is also linked to glutathione metabolism, has been shown to be implicated in the response of macrophages to LPS/IFN‐γ and IL‐4 [39, 40, 41]. In this review, we will discuss how the metabolism of specific amino acids fundamentally regulates macrophage activation and intrinsically controls their function. This article complements a series of recently published reviews focusing solely on TCA cycle metabolites and their role in macrophage polarization as well as on amino acid metabolism in the context of immunity in general [42, 43].

## Classical activation (M1) of macrophages and metabolic adaptation during polarization

Cells belonging to the monocyte/macrophage lineage display a distinct phenotypic heterogeneity, which results from differences in cellular differentiation state, tissue distribution and predominance of a variety of endogenous and exogenous stimuli [44]. In the context of a type 1 effector response, macrophages acquire a phenotype, which mediates immunity to many microorganisms including bacteria, viruses, fungi as well as protozoa and also helps to maintain tumour immune surveillance. The term ‘classical’ immune activation of macrophages that has later also been described as M1 polarization stems from early studies with mice infected with *Listeria monocytogenes* or *M. tuberculosis* [45]. The observation of enhanced antimicrobial activities of macrophages, which can induce resistance to these bacterial infections, was subsequently attributed to cytokines, in particular interferon‐γ (IFN‐γ), that are secreted by specifically activated Th_1_ cells and natural killer cells [46].

In parallel to the observation that macrophages alter their arginine metabolism to produce large amounts of NO when they encounter LPS [18], it was realized that glycolysis is another metabolic pathway, which is substantially upregulated during classical macrophage polarization [47, 48]. Before HIF‐1α was discovered as a strongly activated transcription factor in macrophages following LPS stimulation, because its loss protects from LPS‐induced mortality in mice [16, 49, 50, 51], HIF‐1α had been identified to be a major driver of glycolytic gene expression [52]. This connection is of particular interest as it partially explains why M1‐polarized macrophages engage in aerobic glycolysis even under normoxic conditions. The question arose, which cellular events trigger HIF‐1α activation and stabilization in these macrophages.

**Fig. 1:**
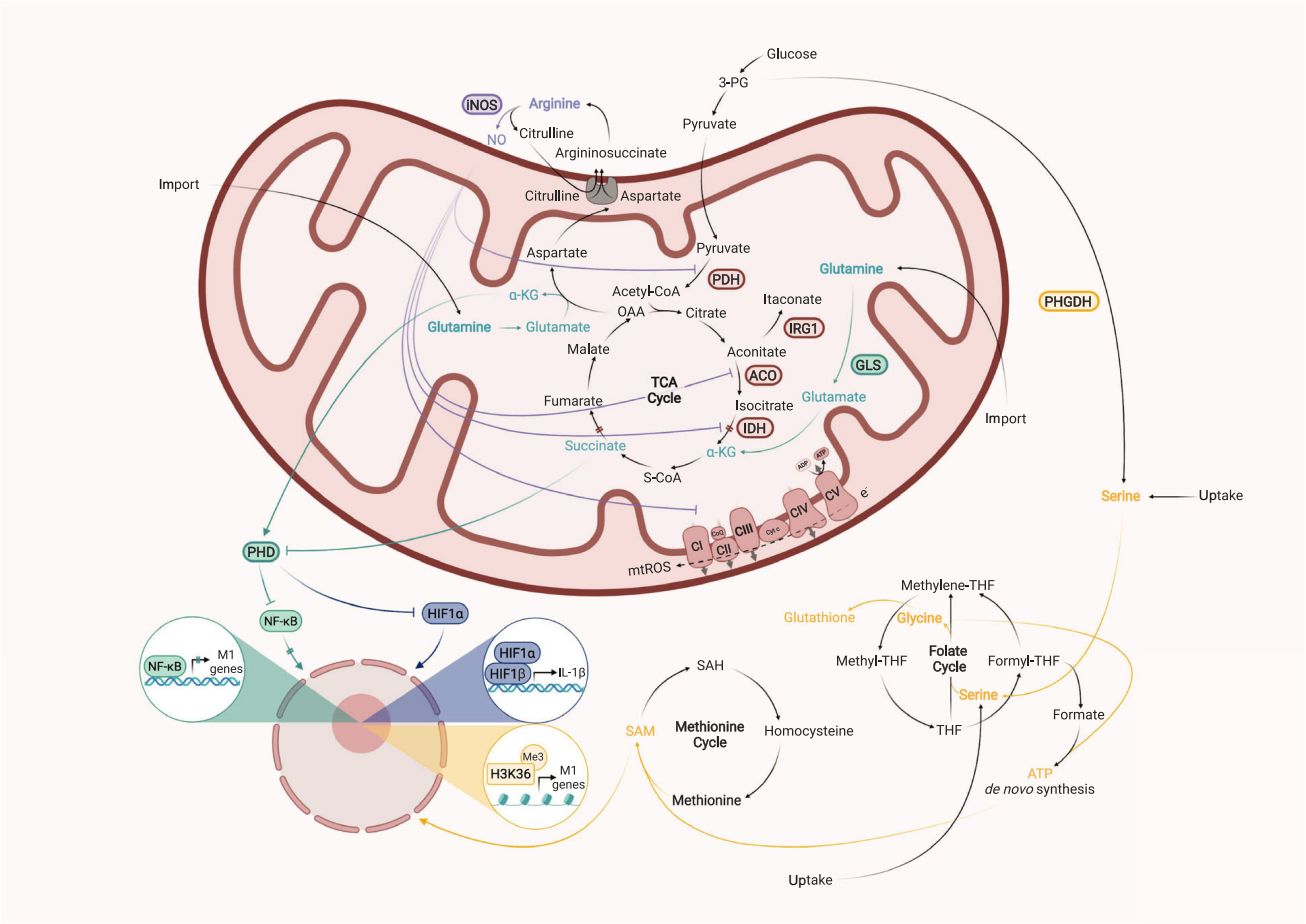
Amino acids and corresponding metabolic pathways shape M1 macrophage activation. The ETC and mitochondrial complexes are remodelled in response to LPS/IFN‐γ stimulation to increase mitochondrial ROS production and decrease respiration. Mitochondrial ROS has been shown to drive the inflammatory response of M1‐polarized macrophages. Arginine is converted via iNOS to citrulline and NO, which inhibits mitochondrial complex I and complex II and promotes the loss of mitochondrial complexes during late stages of M1 polarization. The TCA cycle also undergoes rapid changes during M1 polarization. In the early stage, production of itaconate leads to accumulation of succinate by inhibition of SDH, which stabilizes HIF‐1α and augments the inflammatory response. NO inhibits ACO2 and IDH, which leads to decreased carbon flux from citrate to α‐KG and, which triggers increased carbon entry from glutamine into the TCA cycle to fuel α‐KG and succinate anaplerosis. The ratio of succinate and α‐KG regulates M1 polarization via PHD‐dependent proline hydroxylation of IKKβ, which is important for activation of NF‐κB signalling. High levels of succinate in the early phase of M1 polarization favour a strong inflammatory response. The later phase is characterized by inhibition of PDHC and OGDC, which leads to a drastic reduction in the levels of citrate, itaconate and succinate. PDHC has been shown to be inhibited by NO‐mediated nitrosation of cysteine residues from DLD, a subunit, which also associates with OGDC. Thus, it is also likely that NO mediates the observed diminished flux from α‐KG to succinate in the late phase. Additionaly, glutamine‐derived α‐KG is important for the induction of endotoxin tolerance and GLS inhibition leads to increased mortality in the context of repeated LPS administrations. The SSP is also involved in M1 polarization by generation of glycine and driving the folate cycle, which is important for glutathione and SAM production. Glutathione production is important for optimal IL‐1β expression and SAM promotes H3K36 trimethylation of inflammatory genes to increase their expression.

Besides a mechanism that involves pyruvate kinase M2 [53], another metabolic key event in classically activated macrophages is the accumulation of several TCA cycle intermediates, which is based on a profound change in this metabolic pathway (Fig. 1) [34]. The build‐up of succinate stabilizes HIF‐1α by impairing prolyl hydroxylases (PHDs), which under active conditions hydroxylate HIF‐1α that is further tagged with ubiquitin to induce proteasomal degradation. The decrease in PHD activity is explained by product inhibition. These enzymes use α‐KG to hydroxylate their target proteins, while succinate, the product of this reaction, negatively regulates their function [54]. Furthermore, in 2011 two independent studies reported the discovery of significant accumulation of itaconate in LPS/IFN‐γ‐stimulated macrophages and diseased lung tissue of *M. tuberculosis*‐infected mice [55, 56]. Itaconate is produced via the decarboxylation of cis‐aconitate by aconitate decarboxylase 1 (ACOD1). It took two more years to unravel that immunoresponsive gene 1 (*Irg1*), which had been described already years before as one of the most highly upregulated genes under pro‐inflammatory conditions, encodes for the enzyme ACOD1 [57, 58, 59, 60]. Due to its inhibitory properties on succinate dehydrogenase (SDH), itaconate is involved in the aforementioned accumulation of succinate [61, 62]. Interestingly, Seim *et al*. [63] have shown that itaconate itself can inhibit the activity of PHDs, which together with succinate might lead to the stabilization of HIF‐1α. However, the stabilization of this transcription factor can also be mediated by the oxidation of succinate via SDH. This reaction has been shown to drive reactive oxygen species (ROS) production by hyperpolarization of mitochondria in LPS‐activated macrophages, which induces the production of pro‐inflammatory cytokines such as IL‐1β [64]. In this setting, itaconate would act as a rheostat by inhibiting SDH activity and consistently *Irg1* ^−/−^ macrophages exhibit a more pro‐inflammatory state [61]. Therefore, it is a yet unresolved question, why itaconate exerts anti‐inflammatory properties while at the same time it promotes the build‐up of succinate, which is thought to drive a pro‐inflammatory response. One hypothesis that might solve this controversy is that the effect of mitochondrial ROS production by oxidation of succinate on HIF‐1α stabilization is dominant over the effect of PHD inhibition via succinate and itaconate accumulation. In support of this notion, the OCR of *Irg1* ^−/−^ macrophages is highly increased upon LPS stimulation and *in vivo* administration of itaconate leads to a fast reduction in oxygen uptake of rats [61, 65]. In this regard, the role of succinate as a strictly pro‐inflammatory metabolite has also recently been challenged by several studies, which demonstrate that succinate can have context‐dependent anti‐inflammatory properties [66, 67, 68, 69]. Moreover, it still needs to be defined, which effects derive from the intracellular build‐up of succinate pools during M1 polarization and which from extracellular succinate that can be sensed via succinate receptor 1 (SUCNR1). While an earlier study has demonstrated increased IL‐1β production via SUCNR1 signalling [70], more recent reports propose an anti‐inflammatory role of the succinate‐SUCNR1 axis in tumour‐associated and adipose tissue macrophages [66, 67]. Adding another layer of complexity, the expression of SUCNR1 is variable among the different macrophage polarization states and is predominantly expressed in pro‐resolving or anti‐inflammatory macrophages. Furthermore, signalling via this receptor seems to activate HIF‐1α, but instead of inducing pro‐inflammatory genes it leads to the induction of anti‐inflammatory modulators such as ARG1 [66, 67, 71].

Interestingly, a recent study from Harber *et al*. could not reproduce the effects from previous studies, which demonstrated that adding succinate to *in vitro* classical macrophage activation assays drives the inflammatory response and instead showed that addition of this metabolite or expression of SUCNR1 reduces the induction of M1 marker genes and the production of inflammatory cytokines [69]. Taken together, although the underlying mechanisms are incompletely understood, these studies provide evidence that itaconate and succinate serve important functions for the regulation of macrophage polarization and that the remodelling of the TCA cycle is fundamentally linked to their function.

## Arginine‐derived nitric oxide is involved in the dynamic remodelling of the mitochondrial electron transport chain during M1 polarization

Early work already established that macrophages alter their metabolism by increasing glucose uptake and lactate production while decreasing oxygen consumption when encountering an inflammatory stimulus [72, 73, 74]. A first possible connection between this distinct metabolic phenotype and the short‐lived arginine‐derived metabolite NO was presented by Drapier *et al*. [75, 76]. They showed that the activity of the mitochondrial complexes I (NADH‐Q oxidoreductase) and II (SDH) decrease in LPS/IFN‐γ‐activated macrophages in parallel with the induction of arginine‐derived NO and also noted a concomitant increase in glycolytic activity (Fig. 1). The authors suggested that glycolysis must be upregulated in order to meet the demand for ATP when mitochondrial metabolism is compromised. Subsequently, another effect of NO on the electron transport chain (ETC) was described as it was shown that short‐term exposure to NO reversibly inhibits complex IV, which is competitive with oxygen at the catalytic site of the enzyme [77, 78]. Furthermore, Clementi *et al*. [79] clarified the already established mechanism of complex I inhibition by demonstrating that S‐nitrosylation of this complex can lead to persistent defects of cellular respiration.

Recent studies have explored the role of the ETC with a particular focus on complex I and complex II (SDH) as well as ROS generation during inflammatory responses of macrophages. Deletion of *Ndufs4*, which causes a dysfunctional complex I, increases mitochondrial ROS and upregulates expression of the inflammatory genes *Il1b* and *Tnfa* already in the absence of direct activation [80]. In line with this, it has been shown that the engagement of the Toll‐like receptors TLR1, TLR2 and TLR4 leads to increased ROS production via ubiquitination of the protein evolutionarily conserved signalling intermediate in Toll pathways (ECSIT), which is involved in assembly of mitochondrial complex I [81, 82]. Underlining the hypothesis that ROS induce a more pro‐inflammatory state, chemical inhibition of complex I or expression of the alternative oxidase (AOX) impairs ROS production and leads to reduced IL‐1β levels in LPS‐activated macrophages [83, 84]. Recently, Garaude *et al*. [85] have also demonstrated that upon activation of Toll‐like receptor signalling and the NLRP3 inflammasome, macrophages transiently decrease assembly of complex I‐ and complex I‐containing supercomplexes, which is accompanied by an increase in the activity of complex II. Treatment of mice with the complex II inhibitor dimethyl malonate reduces IL‐1β levels when challenged with viable *E. coli* bacteria. These studies support a model where the ETC of macrophages during M1 polarization undergoes a rapid reorganization, which causes an increase in ROS production driven by changes in the activity of complex I and complex II leading to a pro‐inflammatory state. In the context of this dynamic remodelling of the ETC from classically activated macrophages, a study using *Nos2* ^−/−^ macrophages shows that NO also promotes the loss of mitochondrial ETC complexes in addition to its well‐known inhibitory effect on their function [86] (Fig. 1). Concerning the consequences of the NO‐mediated inhibition of the ETC, Van den Bossche *et al*. [87] provide evidence that M1 macrophages fail to repolarize to an M2‐like phenotype, because M2 polarization is highly dependent on a functional ETC. This lack of polarization plasticity can be partially restored by chemical inhibition of iNOS or in *Nos2* ^−/−^ macrophages. This could also explain previous *in vivo* findings in *Nos2* ^−/−^ mice where preserved plasticity of pro‐atherogenic M1 cells to inflammation‐resolving, pro‐fibrotic M2 macrophages may underlie the decreased atherosclerosis and increased atherosclerotic collagen deposition in these mice [88, 89]. Several questions concerning the specific effects of NO, which modulate the inflammatory state of macrophages, still remain open. If NO inhibits complex II and oxidation of succinate through this complex drives hyperpolarization of mitochondria, which leads to elevated ROS levels, the lack of complex II inhibition in *Nos2* ^−/−^ macrophages would be one possible mechanism explaining the increased levels of pro‐inflammatory cytokines reported in two recent studies [86, 90]. In support of this hypothesis, McNeill *et al*. demonstrate that *Gch1^−/−^* macrophages, which express normal iNOS protein levels but lack NO production, exhibit higher cellular ROS production [91]. Furthermore, ROS production is induced earlier than NO [63], which suggests that the remodelling of the ETC that initiates mitochondrial ROS formation is NO‐independent but sustained NO levels at later time points have a regulatory function by its known inhibition of mitochondrial complexes.

In connection with the proposed compensatory upregulation of glycolysis due to NO‐mediated repression of mitochondrial respiration, it is important to mentioned that two studies did not observe differences in the glycolytic rate between *Nos2* ^−/−^ and wild‐type macrophages and one study even reported higher levels with chemical iNOS inhibition [86, 87, 90]. In two of these studies, extracellular acidification rate was used to derive the glycolytic rate [87, 90]. It has been shown that the extracellular acidification rate can also be highly dependent on acidification from other sources than lactate such as TCA cycle‐derived production of CO_2_ and therefore is not always a reliable parameter for glycolytic flux [92]. In this regard, Bailey *et al*. [90] also reported reduced lactate secretion in *Nos2* ^−/−^ macrophages. Thus, there is conflicting evidence concerning the compensatory upregulation of glycolysis mediated by NO‐derived inhibition of mitochondrial respiration. Another question is whether a similar time‐resolved regulation depending on the late LPS‐induced expression of iNOS also exists in macrophages. This has been shown in dendritic cells, where only the sustained commitment towards a more glycolytic state is mediated by NO and not the rapid early activation upon stimulation with LPS [93, 94].

## The TCA cycle undergoes vast changes during M1 polarization: a key role of arginine‐derived nitric oxide

In parallel to the aforementioned reorganization of the ETC, the TCA cycle also rapidly changes in macrophages encountering a pro‐inflammatory stimulus. Recently, Seim *et al*. [63] have shown that the remodelling of this central metabolic pathway can be divided into two stages. During the early stage, IDH1/2 and aconitase 2 (ACO2) are inhibited or downregulated and the upstream metabolites citrate/isocitrate accumulate. In connection with the rapid induction of ACOD1, these events lead to a diversion of the carbon flux to itaconate. Itaconate competitively inhibits SDH, which results in accumulation of succinate. As a consequence of the succinate build‐up, HIF‐1α levels increase via inhibition of PHD. This event indicates the transition from the early to the late phase metabolic rewiring, which is characterized by upregulation of NO synthesis and activation of the aspartate argininosuccinate shunt that connects anaplerosis of the TCA cycle metabolites fumarate, malate and oxaloacetate with NO synthesis. Furthermore, substantial inhibition of pyruvate dehydrogenase complex (PDHC) that decarboxylates pyruvate to generate acetyl‐CoA leads to reduced carbon entry from glycolysis to the TCA cycle. This results in the reduction of glucose‐derived citrate, cis‐aconitate and itaconate. Simultaneously, inhibition of oxoglutarate dehydrogenase complex (OGDC) dampens succinate accumulation. The net effect is a drastic reduction in these metabolites and normalization of HIF‐1α [63].

Insight from several studies suggests an important role for arginine‐derived NO in the rewiring of the TCA cycle. Concerning the reduction in carbon transition from citrate to α‐KG, it has been shown that NO‐mediated nitrosation of cysteine residues of IDH1 decreases the activity of this enzyme [90] (Fig. 1). Another study demonstrated that the decrease in ACO2 expression and activity, which can be observed during M1 polarization, is absent in *Nos2* ^−/−^ macrophages [86]. Findings from earlier studies also suggest that continuously high NO production can destabilize the required Fe–S cluster of this enzyme [75, 95]. In concordance with the abovementioned observations, isotope tracing experiments with uniformly labelled glucose suggest a functional TCA cycle in *Nos2* ^−/−^ macrophages due to the presence of substantial ratios of m + 4, m + 5 and m + 6 for all TCA cycle intermediates, which is completely absent in NO‐producing wild‐type macrophages upon M1 polarization [86]. Thus, NO is a key determining factor of the observed break in the TCA cycle between citrate and α‐KG.

NO is furthermore involved in the metabolic rewiring at the level of PDHC‐mediated flux from glycolysis into the TCA cycle by nitrosation of cysteine residues from dihydrolipoamide dehydrogenase (DLD), which associates with several metabolic enzymes such as PDHC and OGDC [86] (Fig. 1). Nitrosation of this subunit has been shown to reversibly inhibit its activity and could thus explain the severely compromised carbon flux through PDHC, which is a hallmark of the late phase metabolic adaptation of M1‐polarized macrophages [63, 96]. Accordingly, the absence of NO promotes pyruvate oxidation via PDHC because the flux through this enzyme complex, measured by the ratio of m + 2 citrate/m + 3 pyruvate from uniformly labelled glucose, is not downregulated upon LPS/IFN‐γ stimulation in *Nos2* ^−/−^ macrophages and additionally, pyruvate respiration is comparable to unstimulated cells [86]. As DLD associates also with OGDC and is nitrosated by NO, the observed reduced carbon flow through this enzyme complex in the late metabolic phase might as well be largely mediated by NO similar to the mechanism that has been shown for PDHC. This, however, requires further investigation, and to this date, reports which prove this hypothesis are still missing.

To sum up, besides its role as a potent cytotoxic and bacteriostatic metabolite produced by inflammatory macrophages, NO also serves regulatory functions via its effects on the ETC and TCA cycle (Fig. 1). This is in line with the observed phenotype of *Nos2* ^−/−^ macrophages, which express higher levels of pro‐inflammatory cytokines [86, 90]. NO has also been shown to control the immunopathology of tuberculosis by inhibiting NLRP3 inflammasome‐dependent processing of IL‐1β [97]. In addition, supplementation with arginine also increases NO production and improves the clinical outcome of patients with tuberculosis who are undergoing tuberculostatic therapy, which underscores the important role of this amino acid‐derived metabolite in inflammation and immunity [98].

## A break in the TCA cycle leads to increased anaplerosis of TCA cycle intermediates through glutamine, which serves as an important immune rheostat for M1 polarization

Key characteristics of the early remodelling of the TCA cycle during M1 polarization are the decreased carbon transition from citrate to α‐KG and the build‐up of succinate. Consequently, the replenishment of TCA cycle intermediates downstream of citrate is driven by increased carbon flux from glutamine (Fig. 1). This observation has consistently been shown in several recent publications, which demonstrate through isotope tracing experiments with labelled glutamine that the relative distribution of m + 5 labelled α‐KG and m + 4 labelled succinate substantially increases upon LPS stimulation [34, 35, 63, 99]. The increased anaplerosis of succinate is accompanied by an increase in the glutamine transporter *Slc3a2* gene expression and also increased glutamine uptake [34, 86]. A proportion of this larger succinate pool in macrophages in response to LPS is derived from the gamma aminobutyric acid (GABA) shunt, which bypasses the TCA cycle by using glutamine for conversion to glutamate, GABA, succinic semialdehyde and eventually succinate [34]. The irreversible inhibitor of the key GABA shunt enzyme, GABA transaminase, vigabatrin significantly reduces the amount of labelled succinate from glutamine and thus LPS‐induced stabilization of HIF‐1α and secretion of IL‐1β by macrophages [34]. Furthermore, the stimulatory effect of LPS/IFN‐γ to increase carbon entry into the TCA cycle from glutamine is also NO‐dependent, because *Nos2* ^−/−^ macrophages show decreased fractional labelling from glutamine for succinate and citrate [86]. Thus, glutamine is an important substrate for the anaplerosis of succinate in M1‐polarized macrophages and thereby exerts potent effects on the immune response in an inflammatory setting.

Recently, Liu *et al*. demonstrated by using pharmacological glutaminase (GLS) inhibition or glutamine‐deficient medium and cell‐permeable succinate as well as α‐KG derivates that the α‐KG/succinate ratio could be also an important determinant of the inflammatory response of LPS/IFN‐γ‐stimulated macrophages [38] (Fig. 1). Addition of dimethyl α‐KG dampens M1 polarization by interfering with nuclear factor kappa‐light‐chain‐enhancer of activated B cells (NF‐κB) signalling through suppression of IKKβ activation and eventually NF‐κB translocation. This effect seems to be controlled via PHD‐dependent proline hydroxylation on IKKβ because this enzyme is positively regulated by α‐KG but antagonized by succinate. However, the authors do not show how the α‐KG/succinate ratio is specifically modulated by glutamine‐depleted medium or GLS inhibition, which is one of their main proposed mechanisms. There is controversy concerning the use of derivates as it has been shown for example that different itaconate forms can exert effects that are attributed to their modification and not to the metabolite itself. Additionally, the fact that adding diethyl succinate does not antagonize dimethyl α‐KG‐induced impairment of the IKKβ–NF‐κB pathway, warrants a more critical interpretation of the results of this study [100]. Despite these limitations, this report adds a new perspective of how the metabolic rewiring during M1 polarization could shape the inflammatory outcome. It remains also open, if the α‐KG/succinate ratio changes between the different stages of the metabolic remodelling in LPS/IFN‐γ‐stimulated macrophages, as the downregulation of OGDC during the late stage would reduce the conversion of α‐KG to succinate [63]. Another interesting observation of the study presented by Liu *et al*. is that glutamine starvation or GLS inhibition with BPTES during the LPS priming phase prevents macrophages to become endotoxin tolerant [38]. Endotoxin tolerance is a mechanism, which renders macrophages unresponsive to repeated stimulation by LPS and thus represents a crucial homeostatic mechanism that protects the organism from potentially harmful excessive activation of the immune system [101]. In a toxic shock mouse model with a low priming dose of LPS and a second administration of a lethal dose of LPS, injection of BPTES reduced survival of these mice, whereas the addition of dimethyl α‐KG reverted this phenotype. The exact mechanism of how glutamine metabolism is involved in the induction of endotoxin tolerance, however, still needs to be explored. Recently, DNA demethylase Tet2, which contains binding sites for α‐KG, critically required for its catalytic function, has been shown to also control pro‐inflammatory responses in macrophages [102]. In this respect, it has been proposed that epigenetic reprogramming forms the basis of innate immune memory. Accumulation of fumarate, due to glutamine replenishment of the TCA cycle, induces epigenetic reprogramming of monocytes by inhibiting KDM5 histone demethylases, which is essential for establishing β‐glucan‐induced trained immunity [103, 104, 105, 106]. From a clinical perspective, it is noteworthy that patients with obesity or diabetes have lower serum concentrations of glutamine and α‐KG but higher concentrations of succinate in line with the known accumulation of M1 macrophages in these metabolic disorders [107, 108, 109, 110].

In summary, the replenishment of succinate via increased entry of glutamine‐derived carbon into the TCA cycle is induced during the early stage of the metabolic remodelling in M1 polarization. Furthermore, glutamine‐derived α‐KG seems to dampen the inflammatory response and is crucial for the induction of endotoxin tolerance.

## Serine and glycine are important for redox balance and epigenetic regulation of pro‐inflammatory genes during M1 polarization

In the 80s and 90s, it has already been described that thioglycollate‐elicited, LPS‐stimulated or zymosan‐challenged macrophages greatly increase their uptake for cystine, which is the oxidized dimer form of the amino acid cysteine, and almost release the same amounts of cysteine and glutathione [111, 112, 113]. However, only recently due to studies, which highlight the relevance of increased ROS levels upon M1 polarization, the regulation of redox homeostasis during inflammatory activation sparked new interest. Glutathione is the major antioxidant that protects cells from ROS and is synthesized by the addition of glycine to γ‐glutamylcysteine, which itself consists of glutamate and cysteine. Although the underlying mechanisms are still incompletely understood, it is widely accepted that the transcription factor nuclear factor erythroid‐2‐related factor 2 (NRF2) is activated in response to oxidative stress and restricts NF‐κB activation in response to LPS partly via regulation of glutathione metabolism [114, 115]. Buthionine sulfoximine, an inhibitor of glutamylcysteine synthetase, which blocks glutathione production, has also been shown to decrease serum levels of IL‐1β in endotoxaemia in rats [116]. Thus, it has been speculated that despite substantially induced ROS levels during M1 polarization, which induce upregulation of inflammatory cytokines, ROS must be held below a certain threshold. Elevated ROS production could lead to a dysregulated cellular redox balance and dampen the inflammatory response by NRF2 activation [82, 83, 117]. The relevance of the serine synthesis pathway (SSP) is still poorly defined for immunity and inflammation. However recently, it has been attributed to an unexpected role during M1 polarization [41]. The entry step of the SSP branches from glycolysis and is catalysed via the enzyme phosphoglycerate dehydrogenase (PHGDH) to produce phosphopyruvate, phosphoserine and eventually serine (Fig. 1). Rodriguez *et al*. [41] demonstrated that environmental but also endogenously synthesized serine via the SSP is required for optimal LPS induction of *Il1b* mRNA expression. The conversion of serine to glycine via serine hydroxymethyltransferase 1/2 is coupled with the generation of 5,10‐methylenetetrahydrofolate from tetrahydrofolate. 5,10‐methylenetetrahydrofolate can be used as a one‐carbon donor in multiple reactions including nucleotide synthesis. In their study, the need for one‐carbon donor units as the mechanism underlying the reduced *IL1b* mRNA expression was ruled out because formate, which can provide one‐carbon units, did not rescue the phenotype. Interestingly, the production of glycine from serine seems to be required for glutathione production, because exogenously added glutathione restored *IL1b* mRNA expression in the absence of serine. The authors further demonstrated the relevance of the SSP for the inflammatory response to LPS by showing that injections of a PHGDH inhibitor in a toxic shock model reduced mortality and IL‐1β levels in the circulation, a finding, which has recently been confirmed by another study [40]. An essential question for future research is why the decrease in IL‐1β is dependent on extracellular serine *in vitro*, but dependent on *de novo* serine synthesis *in vivo*. The study also does not explain why monocytes require PHGDH‐dependent serine synthesis *in vivo*, even though serine and glycine are present in their environment. Thus, the precise mechanisms of how the SSP affects IL‐1β are not fully understood. Another in parallel published study supports the hypothesis that the SSP is important for fuelling *de novo* ATP synthesis to drive S‐adenosyl methionine (SAM) generation in inflammatory macrophages [40] (Fig. 1). Isotope tracing experiments with uniformly labelled glucose or serine demonstrate that upon LPS activation macrophages increase their carbon flux through the SSP and one‐carbon metabolism. This induces a high SAM/S‐adenosyl homocysteine ratio, which promotes LPS‐stimulated production of IL‐1β by supporting H3K36 trimethylation to act as a ‘methyl sink’ that promotes transcription elongation and splicing required for the optimal expression of diverse inflammatory genes [118]. Another recently published report finds that chemical inhibition of PHGDH increases levels of IL‐1β, which contradicts the results of the abovementioned studies [39]. Again, it is important to note that the metabolic reprogramming of M1 polarization can be separated in several stages and conflicting results between these studies might be explained by differences in timing as Rodriguez *et al*. [41] and Yu *et al*. [40] focused on early, whereas Wilson *et al*. [39] focused on later time points after LPS stimulation.

In conclusion, the amino acids serine, glycine and their related metabolic pathways, SSP and one‐carbon metabolism, have recently been shown to be involved in the inflammatory response and highlight an emerging theme in the immunometabolism field.

## Alternative (M2) activation of macrophages and metabolic adaptation during polarization

Alternatively activated macrophages are key players in type 2 immunity and therefore important for effective immune responses against large extracellular parasites, for example helminths. Furthermore, they have been shown to be involved in a variety of other physiological and pathological settings, including tissue homeostasis and repair, malignancy, hypersensitivity, allergy and fibrosis [119]. The term ‘alternative macrophage activation’ was first introduced in 1992 by a study demonstrating that IL‐4‐treated, in contrast to IFN‐γ stimulated, peritoneal macrophages upregulate the expression and activity of the mannose receptor (CD206) [120, 121].

In line with previously published data describing an IL‐4‐mediated restriction of specific MHC class II antigen expression and reduced pro‐inflammatory cytokine secretion [122, 123, 124, 125], the authors concluded that IL‐4 initiates an alternative macrophage polarization state [120, 121]. Although this classification nicely correlated with the previously established phenotypes of Th_1_/Th_2_ cells that are potent secretors of IFN‐γ and IL‐4, respectively [126], it was only in the early 21st century when Mills *et al*. introduced the term ‘M1/M2 macrophage’ [127]. In 1995, IL‐13 was identified as another T‐cell‐derived cytokine that shares certain functional characteristics with IL‐4 [128]. Over the years, other alternative macrophage‐activating factors were described, thus leading to the definition of various M2 subcategories based on their respective inducing molecules and on the differential expression of chemokine receptors: M2a (IL‐4/IL‐13), M2b (combined exposure to immune complexes and TLR or IL‐1R agonists) and M2c (IL‐10) [129]. However, since macrophages display a great heterogeneity and express overlapping markers, it is difficult to subdivide them by this strict systematization. Especially *in vivo,* macrophages face a complex range of different environmental stimuli that rather result in a continuum of polarization states, than in a few distinct well‐defined subsets [130].

Nevertheless, some sort of classification of macrophage fates is still widely used by researchers. The best studied model to induce M2‐like macrophage polarization is the stimulation with IL‐4 and/or IL‐13, which leads to the transcription of the well‐known M2 marker genes *Arg1* and resistin‐like molecule‐alpha (*Relmα*) [119, 131]. Importantly, ARG1 was the first enzyme described to be differentially regulated in mac

rophages stimulated with either Th_1_‐ or Th_2_cell‐derived cytokines. These early studies have already suggested the existence of two competitive metabolic states in murine macrophages [22, 23, 24]. They further concluded that this might be of significant biological relevance, listing confirmatory studies describing the reciprocal regulation of iNOS and ARG1 expression in macrophages during tumour rejection and progressive tumour growth as well as in wound healing [24, 132, 133]. Additionally, these studies raised the question about the functional role of ARG1 expression in alternatively activated macrophages and introduced two not mutually exclusive possibilities: since ARG1 and iNOS compete for the same substrate, arginine, a high expression and activity of ARG1 could suppress NO production; on the other hand, the conversion of arginine by ARG1 results in the build‐up of ornithine, the precursor metabolite for polyamine synthesis, which could have a crucial impact on the macrophage polarization state [24]. Until now, the exact role of ARG1 and its substrate in macrophage activation and function is still not completely clarified.

Accumulating data about ARG1 expression triggered by signalling pathways that are usually attributed to the induction of a more pro‐inflammatory macrophage fate further invigorate research about functional consequences of this enzyme in macrophage polarization. In this regard, El Kasmi *et al*. [27] described that TLR signalling that is well known to augment the bactericidal activity of myeloid cells stimulates ARG1 expression in *M. tuberculosis‐* and *Toxoplasma gondii*‐infected macrophages. Importantly, the authors could show that this increase in ARG1 levels is independent of the transcription factor STAT6 but instead mediated by MyD88 signalling. Additionally to this described mechanism of cell‐intrinsic MyD88 signalling, another function of this pathway is the induction of the release of specific cytokines including IL‐6, IL‐10 and G‐CSF. These cytokines in turn activate the transcription factor STAT3 and induce ARG1 expression in an autocrine/paracrine manner [29]. In 2014, a study demonstrated that macrophages stimulated with tumour‐conditioned media display a phenotype similar to cells cultured under anaerobic conditions. The authors identified that lactic acid secreted by tumour cells skews macrophages towards *Arg1* and *Vegfa* expression via increased HIF‐1α stabilization [31], a signalling pathway believed to be only involved in M1 macrophage polarization and that is generally known to be activated via hypoxia [34]. Indeed, a previous study using a *M. tuberculosis* infection disease model has already suggested that an oxygen scare environment might increase ARG1 levels in macrophages [32]. Importantly, perturbation in oxygen homeostasis is also considered a key feature of solid tumours and is associated with macrophage recruitment as well as with the induction of angiogenetic genes [134, 135]. A very recent study by Carmona‐Fontaine *et al*. could demonstrate that ARG1‐expressing macrophages are indeed predominantly located within hypoxic regions of the tumour and simultaneously express VEGFA to stimulate endothelial cells to engage in vessel formation [33]. However, in contrast to the study by Colegio *et al*. [31] the authors state that limited oxygen and high levels of lactate synergistically increase ARG1 expression by TLR signalling and the mitogen‐activated protein kinase (MAPK) pathway. Their findings nicely fit to previously published data that link MyD88 signalling to macrophage ARG1 expression in two different intracellular infection models [27, 29]. Another recent study provides evidence for an epigenetic regulation of ARG1 expression in LPS/IFN‐γ‐stimulated or bacteria‐challenged macrophages by lactate [30]. The authors could show that this metabolite, which accumulates due to an increased glycolytic metabolism of M1‐polarized macrophages, is used for histone lactylation, which in turn activates the transcription of specific genes that are predominantly involved in wound healing and keeping tissue homeostasis. Since histone lactylation happens in the late phase of M1 polarization and mainly stimulates the expression of homeostatic or M2‐associated genes such as ARG1, the authors suggest that lactate might function as a clock to turn off the M1 programme of macrophages. This could also explain the presence of ARG1/iNOS double‐positive myeloid subsets in different disease settings where the immune response might undergo a switch from a pro‐inflammatory to a rather anti‐inflammatory state [26, 27, 28]. Moreover, this study provides evidence for an indirect effect of HIF‐1α on the induction of ARG1 expression in M1 macrophages by increasing the production of lactate through activation of glycolytic genes [30]. Additionally, HIF‐1α might trigger a positive feedback loop, which boosts lactate production via the activation of pyruvate dehydrogenase kinase (PDK) that blocks PDHC and thus enhances the conversion of pyruvate to lactate and therefore ultimately stimulates ARG1 expression in an indirect way via histone lactylation. Taken together, many different pathways that are considered being pro‐inflammatory can lead to the induction of ARG1 levels in macrophages. Thus, further research is needed to clarify the function of this enzyme and its substrate arginine in various macrophage polarization states.

**Fig. 2:**
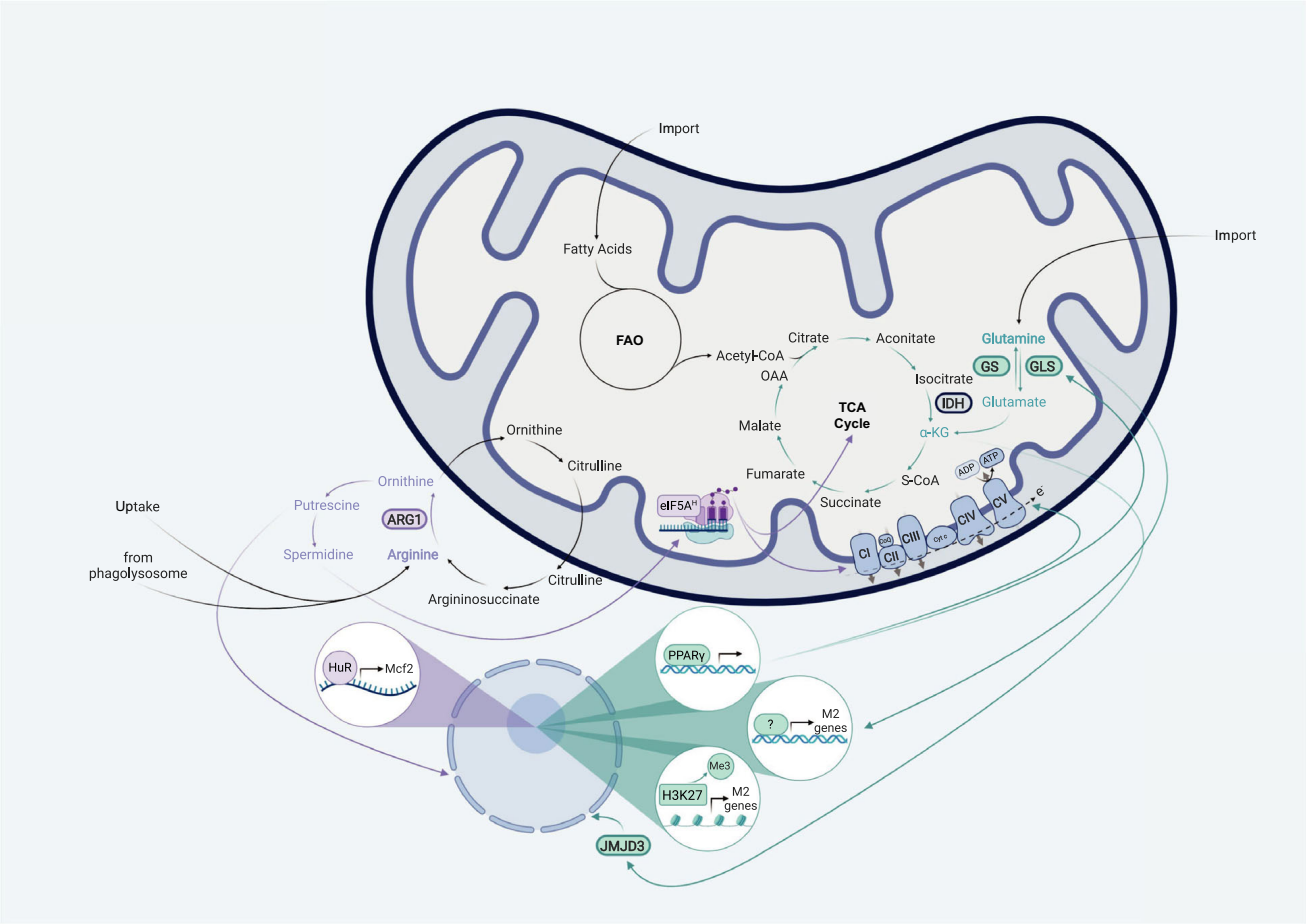
Amino acids and corresponding metabolic pathways shape M2 macrophage activation. In comparison to M1 macrophages, M2‐polarized cells exhibit an intact TCA cycle and enhanced OXPHOS. Furthermore, it is suggested that they prefer glutamine and fatty acids as energy sources in contrast to glucose. Thus, alternatively activated macrophages do not rely on glycolysis as long as they can fuel the TCA cycle with other substrates, for example glutamine. One hallmark of M2 macrophages is the expression of ARG1. This enzyme converts arginine to ornithine, therefore providing the substrate for subsequent polyamine synthesis. Interestingly, intracellular arginine is not only derived from simple environmental uptake, but can also be generated from phagocytosed apoptotic cells in the lysosomes. Its downstream metabolite putrescine leads to HuR‐mediated stabilization of Mcf2 mRNA, a guanine nucleotide exchange factor that activates Rac1. Subsequently, this results in increased actin polymerization and enhanced uptake of apoptotic bodies, a hallmark M2 macrophage function. Additionally, arginine‐derived spermidine is involved in the hypusination of eIF5A, a translation factor, that leadsto the efficient expression of proteins involved in the TCA cycle and in OXPHOS. Another important amino acid for macrophage alternative polarization is glutamine. This nutrient can serve as the precursor metabolite for α‐KG by glutaminolysis (enzymatic reactions involving GLS) that in turn regulates JMJD3‐mediated histone demethylation and thus the expression of M2‐associated genes. Furthermore, α‐KG can feed into the TCA cycle and thereby support enhanced OXPHOS. However, glutamine production via GS might also be critically involved in alternative macrophage activation. Therefore, glutamine per se could influence the phenotype of macrophages by yet unknown mechanisms. For example, this amino acid could serve as a nitrogen source for UDP‐GlcNac, which acts as a substrate for posttranslational modifications of essential M2 marker proteins such as CD206. Another important factor in alternative activation is PPAR‐γ signalling. PPAR‐γ is not only linked to a switch to glutamine metabolism, but also involved in upregulating OXPHOS.

Over the last decade of research, the link between macrophage polarization and metabolism in general has gained more and more attention. In this regard, in 2006 Vats *et al*. [36] demonstrated that alternatively activated macrophages take up more fatty acids and prefer fatty acid oxidation (FAO) over glycolysis in comparison to macrophages stimulated with LPS/IFN‐γ (Fig. 2). The authors further introduced peroxisome proliferator‐activated receptor‐gamma (PPAR‐γ) coactivator 1β (PGC‐1β), as an IL‐4‐dependent STAT6‐mediated transcriptional coactivator that induces the expression of genes associated with FAO and mitochondrial biogenesis, thus linking oxidative metabolism with the M2 polarization state. More recently, it has been demonstrated that this alternative programme of macrophages is not only hallmarked by increased FAO, but also by an intact TCA cycle and enhanced OXPHOS (Fig. 2) [35]. Furthermore, the M2 subtype exhibits increased expression of the lipid scavenger receptor CD36 that mediates the endocytosis of triacylglycerol substrates resulting in cell‐intrinsic lysosomal lipolysis. This in turn provides a source of fatty acids to meet the high demand of these nutrients for elevated levels of OXPHOS, a crucial metabolic pathway for M2 polarization [36, 37]. Apart from that, several studies suggested that alternatively activated macrophages additionally display an enhanced usage of glycolysis, a key metabolic pathway of the M1 programme [136, 137, 138]. A downregulation of M2‐associated markers including RELMα, programmed cell death 1 ligand‐2 (PD‐L2), ARG1 and macrophage galactose N‐acetyl‐galactosamine‐specific lectin 2 (*Mgl2*) as well as an impairment of M2‐mediated functions such as mounting an immune response against parasite infections was demonstrated in two studies by treating macrophages *in vitro* and mice with 2‐deoxyglucose (2‐DG), a well‐established glycolysis inhibitor [136, 137]. The authors stated that enhanced glycolysis is essential for alternative macrophage activation, arguing that acetyl‐CoA derived from pyruvate, the end product of glycolysis, might either be an important substrate for histone acetylation that supports M2 polarization or/and for fatty acid synthesis that fuels FAO and thus promotes the M2 activation programme [136, 137]. However, a very recent study elucidated that 2‐DG exerts several off‐target effects, resulting in the concomitant impairment of glycolysis, OXPHOS and the JAK‐STAT6 pathway by the reduction of intracellular ATP levels that are crucial for the activation of JAK. Thus suggesting that glycolysis is not required for the M2 state as long as OXPHOS remains intact by other fuels such as glutamine [139].

## Much more than a plain M2 marker gene: ARG1 converts arginine to polyamines, which are important for M2 polarization and effector functions

Despite some evidence of ARG1 expression under pro‐inflammatory settings [27, 29, 30, 31, 32, 33], this enzyme that is the key modulator of arginine metabolism in M2 macrophages is generally used as a hallmark gene of the alternative immune response. Many studies have tried to elucidate the impact of this substrate–enzyme interaction in the context of various diseases [140, 141]. For example, in *M. tuberculosis* infection the depletion of arginine via ARG1 was suggested to compete with and suppress iNOS activity, thus inhibiting the iNOS‐dependent macrophage effector functions against *M. tuberculosis* [142]. Similarly, the reduction in arginine availability for Th_2_ cell responses in schistosomiasis has been described and linked to alternatively activated macrophages [143]. Furthermore, products of arginine, including ornithine and polyamines, might directly impact pathogen fitness in a beneficial or detrimental manner [144, 145].

Despite all these examples of how arginine and its downstream metabolites influence macrophage responses and disease outcomes, the exact fate of arginine in M2 macrophages for cell‐intrinsic signalling purposes still needs more investigation. In this regard, two very recently published studies shed light on polyamine synthesis in the context of macrophage polarization and function. In general, polyamines are involved in a great variety of cellular processes, including proliferation, autophagy, binding DNA and modulating ion channels [146, 147]. Although polyamines may also form independently of ARG1 in macrophages [148], the main pathway of polyamine synthesis uses arginine as the core substrate that is further metabolized via subsequent enzymatic reactions, including ARG1 (Fig. 2) [149]. IL‐4‐stimulated macrophages have been demonstrated to use this pathway to generate spermidine for the hypusination of the eukaryotic translation initiation factor 5A (eIF5A), which in turn promotes the efficient expression of a subset of mitochondrial proteins involved in the TCA cycle and OXPHOS that are important for M2 polarization (Fig. 2). Although most of the experiments performed to unravel the underlying mechanism were conducted using mouse embryonic fibroblasts, the authors tried to recapitulate the most striking findings in murine bone marrow‐derived macrophages and in an *in vivo* setting of helminth infection by treating animals with GC7, a deoxyhypusine synthase (DHPS) inhibitor, that blocks the hypusination of eIF5A. Furthermore, they performed isotope tracing experiments with labelled arginine to undoubtedly demonstrate that arginine serves as the core substrate for putrescine and most likely spermidine [147]. The second study about polyamines and alternative macrophage polarization focused on efferocytosis in the context of atherosclerosis. The authors could nicely build a link between ingested apoptotic cells and arginine metabolism‐driven alternative macrophage function. In particular, macrophages were described to use arginine and/or ornithine from lysosomal degradation of apoptotic bodies to produce putrescine. This polyamine triggers a positive feedforward loop resulting in enhanced efferocytosis by human antigen R (HuR)‐mediated stabilization of *Mcf2*, the mRNA encoding for proto‐oncogene DBL, a guanine nucleotide exchange factor (GEF) activating Ras‐related C3 botulinum toxin substrate 1 (RAC1) and thus increasing actin polymerization and apoptotic cell internalization (Fig. 2). Importantly, this study could identify arginine derived from ingested cells as the primary source for putrescine by tracing this amino acid into ornithine and putrescine in macrophages that took up Jurkat cells that have been fed labelled arginine [150]. Moreover, it is important to note that although human monocyte‐derived macrophages do not upregulate ARG1 expression upon IL‐4 treatment and thus directly utilize ornithine to engage in continual efferocytosis, ARG1 could be detected in human atherosclerotic plaques, especially in foamy macrophages [150, 151]. Therefore, further studies are needed to clarify the role of arginine and ornithine in human disease settings. In line with the importance of ornithine for alternative macrophage‐mediated immune responses, it was recently demonstrated that ornithine plays a crucial role in *M. tuberculosis*‐infected macrophages. In contrast to macrophages isolated from various other tissues, Kupffer cells are best equipped for killing these bacteria [152]. Importantly, the authors could link the liver‐derived macrophage phenotype to an enhanced intracellular ornithine pool that neutralizes bacteria‐derived ammonium, thus inducing AMP‐activated protein kinase (AMPK) signalling and mediating increased autophagy, a mechanism involved in bacterial clearance [152, 153]. Concluding, although arginine has long been known to be a key amino acid associated with alternative macrophage polarization, the underlying mechanisms of how this nutrient shapes myeloid cell fate are still not fully resolved and thus remain interesting topics to be addressed in future research.

## Glutamine is an essential metabolic regulator of M2 polarization

Investigations concerning the role of metabolic pathways apart from arginine in M2 macrophage polarization began in 2006 with a study establishing the importance of oxidative metabolism for alternative activation [36]. However, it was only in 2015, when researchers conducted parallel unbiased analysis of the transcriptome and metabolome of M1 and M2 macrophages. Integrating these metabolic and transcriptional data did not only validate the notion of an intact TCA cycle in alternatively activated macrophages, but also determined two pathways that had not been discussed in the context of macrophage polarization before: glutamine metabolism and the uridine diphosphate N‐acetylglucosamine (UDP‐GlcNac) pathway (Fig. 2). Using labelled glucose and glutamine tracing experiments, the authors could validate that a third of all carbons in intermediates of the TCA cycle are derived from glutamine. Furthermore, depleting the environment from this amino acid inhibits macrophage activation towards an M2 state in an mTOR‐independent way. Concomitantly to the downregulation of M2‐specific marker genes, TCA cycle‐associated transcripts were significantly reduced in glutamine‐starved IL‐4‐treated macrophages, thus providing a link between macrophage polarization and glutamine metabolism (Fig. 2). Concerning the UDP‐GlcNac pathway, tracing experiments revealed that both glucose and glutamine are a major source for UDP‐GlcNac [35]. As most M2 hallmark proteins including CD206 and RELMα are known to be highly glycosylated [130], the authors suggested that UDP‐GlcNac metabolism may directly impact the alternative macrophage fate by providing the substrate for proper protein folding and trafficking of its signature molecules [35]. Further evidence for the importance of glutamine metabolism regarding alternative activation was provided by a study demonstrating that glutaminolysis derived α‐KG induces metabolic and epigenetic reprogramming in macrophages towards the M2 polarization state. In line with the data from Jha *et al*. [35], Liu *et al*. [38] show that glutamine deprivation leads to a decrease in M2 marker gene expression, ARG1 activity as well as OXPHOS (Fig. 2). Similar impairments in alternative activation could be obtained by inhibiting glutaminolysis via BPTES. Importantly, these changes were independent from a blockage in IL‐4‐mediated STAT6 signalling, thus suggesting alternative underlying mechanisms [38]. Histone H3 lysine‐27 (H3K27) demethylation by Jumonji domain‐containing 3 (JMJD3), a pathway induced downstream of STAT6, has previously been shown to support IL‐4‐induced macrophage priming by promoting the expression of M2‐specific target genes [154]. Liu *et al*. provided evidence that α‐KG serves as a regulator for JMJD3‐mediated histone demethylation by demonstrating that H3K27 methylation marks in glutamine‐deprived macrophages could be removed via adding dimethyl‐α‐KG, a cell‐permeable analogue of α‐KG (Fig. 2) [38]. In line with the crucial role of this metabolite in M2 polarization, compared to healthy controls patients with obesity display reduced serum glutamine and α‐KG levels, suggesting that their macrophages might have deficits in disease‐resolving alternative activation [155]. Moreover, this study provides a possible mechanism for why glutamine supplementation might be beneficial for the recovery after surgery [156]. In parallel, another report claims that the reverse reaction, glutamine production by glutamine synthetase (GS), is also essential for the M2 programme *in vitro* and *in vivo*. Pharmacological inhibition of GS results in a shift from glutamine to glucose utilization for the generation of glutamate and TCA cycle intermediates as well as in an increase of intracellular succinate, a hallmark metabolite of M1 macrophage polarization (Fig. 2). Correspondingly, the authors could detect enhanced expression of genes involved in a pro‐inflammatory response and stabilization of HIF‐1α. Genetic deletion of GS in macrophages in a lung carcinoma model leads to less metastases formation because of the more pro‐inflammatory phenotype of these myeloid cells [157]. Together, this suggests that the impact of glutamine on the M2 macrophage programme might not be solely due to its conversion to α‐KG and subsequent demethylation of H3K27, but could be directly mediated by glutamine itself by a yet unknown mechanism (Fig. 2). It is also important to note that PPAR‐γ signalling is critically involved in glutamine metabolism. Previous studies indicated that PPAR‐γ expression is induced by IL‐4 and plays a role in metabolism, particularly in FAO and mitochondrial biogenesis [36, 158]. Nelson *et al*. [159] provided new insights into PPAR‐γ signalling by connecting it to glutamine metabolism and suggesting a feedforward loop regulating alternative macrophage activation with PPAR‐γ as the nexus. Importantly, it could also be shown that unstimulated macrophages that lack PPAR‐γ exhibit a pro‐inflammatory phenotype and reduced OXPHOS (Fig. 2). Similar findings were presented by a study from Palmieri *et al*., where the inhibition of GS in macrophages results in the accumulation of succinate and an increased stability of HIF‐1α [157]. Moreover, Liu *et al*. [38] established that the ratio of α‐KG to succinate determines the fate of macrophage polarization. Altogether, this suggests that glutamine metabolism is a key regulator of macrophage polarization and might enable macrophages to switch their phenotype according to the environmental stimuli they face.

## Concluding remarks and future perspectives

The M1/M2 model of macrophage polarization has been introduced nearly 30 years ago and is based in part on the expression of the two arginine‐metabolizing enzymes iNOS and ARG1. Since its introduction, this model has not lost its relevance and studies from the last decade even reinforce the notion that these two polarization markers are intrinsically linked to the function of macrophages and dictate their activation state. A lot of progress has recently been made by elucidating the global changes in macrophage metabolism in response to M1 and M2 stimuli, most notably LPS/IFN‐γ and IL‐4/IL‐13. In this respect, the metabolic pathways utilizing other amino acids have emerged as important immunomodulating routes. Additionally to arginine, glutamine due to its ability to replenish TCA cycle metabolites and serine that feeds into the one‐carbon metabolism have been shown to be fundamentally involved in macrophage biology. One of the most crucial features of macrophage polarization is the time‐resolved metabolic rewiring. This has to be taken into consideration for meaningful interpretation of results and planning of conclusive experiments. However, the most important task for future research is to bring the field of metabolically regulated macrophage polarization to a systemic level. More and more studies now aim to characterize distinct tissue niches in respect to metabolite levels and how these might explain the diverse functions of tissue‐resident macrophages versus invading monocytes in the context of inflammation [160, 161, 162]. Therefore, we are only at the beginning to learn about how immunometabolic relationships between cells translate into responses that affect specific organs and eventually the whole organism. Systemic immunometabolism will also lead to a better understanding of how macrophages interact with nonimmune cells and how these relationships are involved in diseases including systemic autoimmune disorders, cancers, metabolic syndrome and infections. Such a crosstalk has been proposed by a very recent report, which suggests that, upon infection, structural nonimmune cells in various organs increasingly interact with immune cells like monocytes, macrophages and B cells [163]. We anticipate that future studies, which connect macrophage activation with systemic immunometabolism, will uncover unexpected consequences of macrophage function in health and disease and hopefully will serve as the basis for innovative therapies to treat a great variety of diseases.

In this regard, it will be crucial to keep the discrepancies in macrophage biology between mouse and human in mind. Particularly, in the context of the applicability of the iNOS/ARG1 dichotomy, conflicting results have been reported, for example that *in vitro* differentiated human macrophages lack the expression of iNOS and ARG1, when treated with stimuli that dramatically induce these enzymes in murine macrophages. However, in diseased tissues from tuberculosis patients and in atherosclerotic plaque robust expression levels of both iNOS and ARG1 have been detected [151, 164]. Therefore, more investigationsare eagerly awaited as these might provide important pieces to solve the ‘polarization puzzle’ between species.

## Conflicts of interest

The authors declare no conflict of interest.

## Author contributions

MK, MH and GS wrote the review. MK and MH designed the figures.

### Peer Review

The peer review history for this article is available at https://publons.com/publon/10.1111/febs.15715.

[Correction added on 19 March 2021, after first online publication: URL for peer review history has been corrected.]

## References

[1] Warburg O, Wind F & Negelein E (1927) The metabolism of tumors in the body. J Gen Physiol 8, 519–530. 19872213 10.1085/jgp.8.6.519 PMC2140820

[2] Warburg O, Gawehn K & Geissler AW (1958) Metabolism of leukocytes. Zeitschrift fur Naturforschung Teil B, Chemie, Biochemie, Biophysik, Biologie und verwandte Gebiete 13b, 515–516. 13593654

[3] Levene P & Meyer G (1912) On the action of leucocytes on glucose second communication. J Biol Chem 12, 265–273.

[4] Levene PA & Meyer GM (1912) THE action of leucocytes on glucose. J Biol Chem 11, 361–370.

[5] Fleischmann W & Kubowitz F (1927) Über den stoffwechsel der leukocyten. Biochem Z 181, 395.

[6] Bakker A (1927) Einige Übereinstimmungen im Stoffwechsel der Carcinomzellen und Exsudatleukocyten. Klin Wochenschr 6, 252–254.

[7] Hedeskov CJ (1968) Early effects of phytohaemagglutinin on glucose metabolism of normal human lymphocytes. Biochem J 110, 373–380. 5726214 10.1042/bj1100373 PMC1187214

[8] Roos D & Loos JA (1970) Changes in the carbohydrate metabolism of mitogenically stimulated human peripheral lymphocytes. I. Stimulation by phytohaemagglutinin. Biochim Biophys Acta 222, 565–582. 5496487 10.1016/0304-4165(70)90182-0

[9] Culvenor JG & Weidemann MJ (1976) Phytohaemagglutinin stimulation of rat thymus lymphocyte glycolysis. Biochim Biophys Acta – Gen Sub 437, 354–363. 10.1016/0304-4165(76)90005-2 1085166

[10] Whitesell RR, Johnson RA, Tarpley HL & Regen DM (1977) Mitogen‐stimulated glucose transport in thymocytes. Possible role of Ca++ and antagonism by adenosine 3':5'‐monophosphate. J Cell Biol 72, 456–469. 188831 10.1083/jcb.72.2.456 PMC2111010

[11] Hume DA, Radik JL, Ferber E & Weidemann MJ (1978) Aerobic glycolysis and lymphocyte transformation. Biochem J 174, 703–709. 310305 10.1042/bj1740703 PMC1185973

[12] Brand K, Williams JF & Weidemann MJ (1984) Glucose and glutamine metabolism in rat thymocytes. Biochem J 221, 471–475. 6332620 10.1042/bj2210471 PMC1144061

[13] Steck TL, Kaufman S & Bader JP (1968) Glycolysis in chick embryo cell cultures transformed by Rous sarcoma virus. Can Res 28, 1611–1619. 4299826

[14] Munyon WH & Merchant DJ (1959) The relation between glucose utilization, lactic acid production and utilization and the growth cycle of L strain fibroblasts. Exp Cell Res 17, 490–498. 13672205 10.1016/0014-4827(59)90069-2

[15] Lunt SY & Heiden MGV (2011) Aerobic glycolysis: meeting the metabolic requirements of cell proliferation. Annu Rev Cell Dev Biol 27, 441–464. 21985671 10.1146/annurev-cellbio-092910-154237

[16] Cramer T, Yamanishi Y, Clausen BE, Förster I, Pawlinski R, Mackman N, Haase VH, Jaenisch R, Corr M, Nizet V*et al*. (2003) HIF‐1alpha is essential for myeloid cell‐mediated inflammation. Cell 112, 645–657. 12628185 10.1016/s0092-8674(03)00154-5 PMC4480774

[17] Mitchell H, Shonle H & Grindley H (1916) The origin of the nitrates in the urine. J Biol Chem 24, 461–490.

[18] Stuehr DJ & Marletta MA (1985) Mammalian nitrate biosynthesis: mouse macrophages produce nitrite and nitrate in response to Escherichia coli lipopolysaccharide. Proc Natl Acad Sci U S A 82, 7738–7742. 3906650 10.1073/pnas.82.22.7738 PMC391409

[19] Hibbs JB Jr, Taintor RR & Vavrin Z (1987) Macrophage cytotoxicity: role for L‐arginine deiminase and imino nitrogen oxidation to nitrite. Science 235, 473–476. 2432665 10.1126/science.2432665

[20] Granger DL, Hibbs JB Jr, Perfect JR & Durack DT (1988) Specific amino acid (L‐arginine) requirement for the microbiostatic activity of murine macrophages. J Clin Invest 81, 1129–1136. 3280600 10.1172/JCI113427 PMC329641

[21] Kung JT, Brooks SB, Jakway JP, Leonard LL & Talmage DW (1977) Suppression of in vitro cytotoxic response by macrophages due to induced arginase. J Exp Med 146, 665–672. 19551 10.1084/jem.146.3.665 PMC2180806

[22] Corraliza IM, Soler G, Eichmann K & Modolell M (1995) Arginase induction by suppressors of nitric oxide synthesis (IL‐4, IL‐10 and PGE2) in murine bone‐marrow‐derived macrophages. Biochem Biophys Res Comm 206, 667–673. 7530004 10.1006/bbrc.1995.1094

[23] Modolell M, Corraliza IM, Link F, Soler G & Eichmann K (1995) Reciprocal regulation of the nitric oxide synthase/arginase balance in mouse bone marrow‐derived macrophages by TH1 and TH2 cytokines. Eur J Immunol 25, 1101–1104. 7537672 10.1002/eji.1830250436

[24] Munder M, Eichmann K & Modolell M (1950) (1998) Alternative metabolic states in murine macrophages reflected by the nitric oxide synthase/arginase balance: competitive regulation by CD4+ T cells correlates with Th1/Th2 phenotype. J Immunol (Baltimore, Md: 1950) 160, 5347–5354. 9605134

[25] Montaner LJ, da Silva RP, Sun J, Sutterwala S, Hollinshead M, Vaux D & Gordon S (1999) Type 1 and type 2 cytokine regulation of macrophage endocytosis: differential activation by IL‐4/IL‐13 as opposed to IFN‐γ or IL‐10. J Immunol 162, 4606–4613. 10202000

[26] Molgora M, Esaulova E, Vermi W, Hou J, Chen Y, Luo J, Brioschi S, Bugatti M, Omodei AS, Ricci B*et al*. (2020) TREM2 modulation remodels the tumor myeloid landscape enhancing Anti‐PD‐1 immunotherapy. Cell 182, 886–900.e17. 32783918 10.1016/j.cell.2020.07.013 PMC7485282

[27] El Kasmi KC, Qualls JE, Pesce JT, Smith AM, Thompson RW, Henao‐Tamayo M, Basaraba RJ, König T, Schleicher U, Koo MS*et al*. (2008) Toll‐like receptor‐induced arginase 1 in macrophages thwarts effective immunity against intracellular pathogens. Nat Immunol 9, 1399–1406. 18978793 10.1038/ni.1671 PMC2584974

[28] Locatelli G, Theodorou D, Kendirli A, Jordão MJC, Staszewski O, Phulphagar K, Cantuti‐Castelvetri L, Dagkalis A, Bessis A, Simons M*et al*. (2018) Mononuclear phagocytes locally specify and adapt their phenotype in a multiple sclerosis model. Nat Neurosci 21, 1196–1208. 30127427 10.1038/s41593-018-0212-3

[29] Qualls JE, Neale G, Smith AM, Koo MS, DeFreitas AA, Zhang H, Kaplan G, Watowich SS & Murray PJ (2010) Arginine usage in mycobacteria‐infected macrophages depends on autocrine‐paracrine cytokine signaling. Sci Signal 3, ra62. 20716764 10.1126/scisignal.2000955 PMC2928148

[30] Zhang D, Tang Z, Huang H, Zhou G, Cui C, Weng Y, Liu W, Kim S, Lee S, Perez‐Neut M*et al*. (2019) Metabolic regulation of gene expression by histone lactylation. Nature 574, 575–580. 31645732 10.1038/s41586-019-1678-1 PMC6818755

[31] Colegio OR, Chu NQ, Szabo AL, Chu T, Rhebergen AM, Jairam V, Cyrus N, Brokowski CE, Eisenbarth SC, Phillips GM*et al*. (2014) Functional polarization of tumour‐associated macrophages by tumour‐derived lactic acid. Nature 513, 559–563. 25043024 10.1038/nature13490 PMC4301845

[32] Duque‐Correa MA, Kühl AA, Rodriguez PC, Zedler U, Schommer‐Leitner S, Rao M, Weiner J 3rd, Hurwitz R, Qualls JE, Kosmiadi GA*et al*. (2014) Macrophage arginase‐1 controls bacterial growth and pathology in hypoxic tuberculosis granulomas. Proc Natl Acad Sci USA 111, E4024–E4032. 25201986 10.1073/pnas.1408839111 PMC4183271

[33] Carmona‐Fontaine C, Deforet M, Akkari L, Thompson CB, Joyce JA & Xavier JB (2017) Metabolic origins of spatial organization in the tumor microenvironment. Proc Natl Acad Sci USA 114, 2934–2939. 28246332 10.1073/pnas.1700600114 PMC5358370

[34] Tannahill GM, Curtis AM, Adamik J, Palsson‐McDermott EM, McGettrick AF, Goel G, Frezza C, Bernard NJ, Kelly B, Foley NH*et al*. (2013) Succinate is an inflammatory signal that induces IL‐1β through HIF‐1α. Nature 496, 238–242. 23535595 10.1038/nature11986 PMC4031686

[35] Jha AK, Huang S‐C, Sergushichev A, Lampropoulou V, Ivanova Y, Loginicheva E, Chmielewski K, Stewart KM, Ashall J, Everts B*et al*. (2015) Network integration of parallel metabolic and transcriptional data reveals metabolic modules that regulate macrophage polarization. Immunity 42, 419–430. 25786174 10.1016/j.immuni.2015.02.005

[36] Vats D, Mukundan L, Odegaard JI, Zhang L, Smith KL, Morel CR, Greaves DR, Murray PJ & Chawla A (2006) Oxidative metabolism and PGC‐1β attenuate macrophage‐mediated inflammation. Cell Metab 4, 13–24. 16814729 10.1016/j.cmet.2006.05.011 PMC1904486

[37] Huang SC‐C, Everts B, Ivanova Y, O'Sullivan D, Nascimento M, Smith AM, Beatty W, Love‐Gregory L, Lam WY, O'Neill CM*et al*. (2014) Cell‐intrinsic lysosomal lipolysis is essential for alternative activation of macrophages. Nat Immunol 15, 846–855. 25086775 10.1038/ni.2956 PMC4139419

[38] Liu PS, Wang H, Li X, Chao T, Teav T, Christen S, Di Conza G, Cheng WC, Chou CH, Vavakova M*et al*. (2017) alpha‐ketoglutarate orchestrates macrophage activation through metabolic and epigenetic reprogramming. Nat Immunol 18, 985–994. 28714978 10.1038/ni.3796

[39] Wilson JL, Nägele T, Linke M, Demel F, Fritsch SD, Mayr HK, Cai Z, Katholnig K, Sun X, Fragner L*et al*. (2020) Inverse data‐driven modeling and multiomics analysis reveals Phgdh as a metabolic checkpoint of macrophage polarization and proliferation. Cell Rep 30, 1542–1552.e7. 32023468 10.1016/j.celrep.2020.01.011 PMC7003064

[40] Yu W, Wang Z, Zhang K, Chi Z, Xu T, Jiang D, Chen S, Li W, Yang X, Zhang X*et al*. (2019) One‐carbon metabolism supports S‐Adenosylmethionine and histone methylation to drive inflammatory macrophages. Mol Cell 75, 1147–1160.e5. 31420217 10.1016/j.molcel.2019.06.039

[41] Rodriguez AE, Ducker GS, Billingham LK, Martinez CA, Mainolfi N, Suri V, Friedman A, Manfredi MG, Weinberg SE, Rabinowitz JD*et al*. (2019) Serine metabolism supports macrophage IL‐1β production. Cell Metab 29, 1003–1011.e4. 30773464 10.1016/j.cmet.2019.01.014 PMC6447453

[42] Kelly B & Pearce EL (2020) Amino Assets: How Amino Acids Support Immunity. Cell Metabolism 32, 154–175. 32649859 10.1016/j.cmet.2020.06.010

[43] Ryan DG & O'Neill LAJ (2020) Krebs cycle reborn in macrophage immunometabolism. Annu Rev Immunol 38, 289–313. 31986069 10.1146/annurev-immunol-081619-104850

[44] Murphy K & Weaver C (2017) Janeway’s Immunobiology, 9th, edn. Garland Science.

[45] Mackaness G (1964) The immunological basis of acquired cellular resistance. J Exp Med 120, 105–120. 14194388 10.1084/jem.120.1.105 PMC2137723

[46] Dalton DK, Pitts‐Meek S, Keshav S, Figari IS, Bradley A & Stewart TA (1993) Multiple defects of immune cell function in mice with disrupted interferon‐gamma genes. Science 259, 1739–1742. 8456300 10.1126/science.8456300

[47] Bustos R & Sobrino F (1992) Stimulation of glycolysis as an activation signal in rat peritoneal macrophages. Effect of glucocorticoids on this process. Biochem J 282 (Pt 1), 299–303. 1311557 10.1042/bj2820299 PMC1130922

[48] Wu G, Field CJ & Marliss EB (1991) Glucose and glutamine metabolism in rat macrophages: enhanced glycolysis and unaltered glutaminolysis in spontaneously diabetic BB rats. Biochim Biophys Acta (BBA) – Gen Sub 1115, 166–173. 10.1016/0304-4165(91)90026-d 1764469

[49] Blouin CC, Pagé EL, Soucy GM & Richard DE (2004) Hypoxic gene activation by lipopolysaccharide in macrophages: implication of hypoxia‐inducible factor 1α. Blood 103, 1124–1130. 14525767 10.1182/blood-2003-07-2427

[50] Peyssonnaux C, Cejudo‐Martin P, Doedens A, Zinkernagel AS, Johnson RS & Nizet V (2007) Cutting edge: essential role of hypoxia inducible factor‐1α in development of Lipopolysaccharide‐Induced Sepsis. J Immunol 178, 7516–7519. 17548584 10.4049/jimmunol.178.12.7516

[51] Frede S, Stockmann C, Freitag P & Fandrey J (2006) Bacterial lipopolysaccharide induces HIF‐1 activation in human monocytes via p44/42 MAPK and NF‐κB. Biochem J 396, 517–527. 16533170 10.1042/BJ20051839 PMC1482811

[52] Iyer NV, Kotch LE, Agani F, Leung SW, Laughner E, Wenger RH, Gassmann M, Gearhart JD, Lawler AM, Yu AY*et al*. (1998) Cellular and developmental control of O2 homeostasis by hypoxia‐inducible factor 1 alpha. Genes Dev 12, 149–162. 9436976 10.1101/gad.12.2.149 PMC316445

[53] Palsson‐McDermott EM, Curtis AM, Goel G, Lauterbach MAR, Sheedy FJ, Gleeson LE, van den Bosch MWM, Quinn SR, Domingo‐Fernandez R, Johnston DGW*et al*. (2015) Pyruvate kinase M2 regulates Hif‐1a activity and IL‐1b induction and is a critical determinant of the Warburg effect in LPS‐activated macrophages. Cell Metab 21, 65–80. 25565206 10.1016/j.cmet.2014.12.005 PMC5198835

[54] Selak MA, Armour SM, MacKenzie ED, Boulahbel H, Watson DG, Mansfield KD, Pan Y, Simon MC, Thompson CB & Gottlieb E (2005) Succinate links TCA cycle dysfunction to oncogenesis by inhibiting HIF‐α prolyl hydroxylase. Cancer Cell 7, 77–85. 15652751 10.1016/j.ccr.2004.11.022

[55] Strelko CL, Lu W, Dufort FJ, Seyfried TN, Chiles TC, Rabinowitz JD & Roberts MF (2011) Itaconic acid is a mammalian metabolite induced during macrophage activation. J Am Chem Soc 133, 16386–16389. 21919507 10.1021/ja2070889 PMC3216473

[56] Shin JH, Yang JY, Jeon BY, Yoon YJ, Cho SN, Kang YH, Ryu DH & Hwang GS (2011) (1)H NMR‐based metabolomic profiling in mice infected with Mycobacterium tuberculosis. J Proteome Res 10, 2238–2247. 21452902 10.1021/pr101054m

[57] Michelucci A, Cordes T, Ghelfi J, Pailot A, Reiling N, Goldmann O, Binz T, Wegner A, Tallam A, Rausell A*et al*. (2013) Immune‐responsive gene 1 protein links metabolism to immunity by catalyzing itaconic acid production. Proc Natl Acad Sci U S A 110, 7820–7825. 23610393 10.1073/pnas.1218599110 PMC3651434

[58] Tangsudjai S, Pudla M, Limposuwan K, Woods DE, Sirisinha S & Utaisincharoen P (2010) Involvement of the MyD88‐independent pathway in controlling the intracellular fate of Burkholderia pseudomallei infection in the mouse macrophage cell line RAW 264.7. Microbiol Immunol 54, 282–290. 20536725 10.1111/j.1348-0421.2010.00205.x

[59] Degrandi D, Hoffmann R, Beuter‐Gunia C & Pfeffer K (2009) The proinflammatory cytokine‐induced IRG1 protein associates with mitochondria. J Interferon Cytok Res 29, 55–67. 10.1089/jir.2008.0013 19014335

[60] Basler T, Jeckstadt S, Valentin‐Weigand P & Goethe R (2006) Mycobacterium paratuberculosis, Mycobacterium smegmatis, and lipopolysaccharide induce different transcriptional and post‐transcriptional regulation of the IRG1 gene in murine macrophages. J Leukoc Biol 79, 628–638. 16415166 10.1189/jlb.0905520

[61] Lampropoulou V, Sergushichev A, Bambouskova M, Nair S, Vincent EE, Loginicheva E, Cervantes‐Barragan L, Ma X, Huang SC, Griss T*et al*. (2016) Itaconate links inhibition of succinate dehydrogenase with macrophage metabolic remodeling and regulation of inflammation. Cell Metab 24, 158–166. 27374498 10.1016/j.cmet.2016.06.004 PMC5108454

[62] Cordes T, Wallace M, Michelucci A, Divakaruni AS, Sapcariu SC, Sousa C, Koseki H, Cabrales P, Murphy AN, Hiller K*et al*. (2016) Immunoresponsive gene 1 and itaconate inhibit succinate dehydrogenase to modulate intracellular succinate levels. J Biol Chem 291, 14274–14284. 27189937 10.1074/jbc.M115.685792 PMC4933182

[63] Seim GL, Britt EC, John SV, Yeo FJ, Johnson AR, Eisenstein RS, Pagliarini DJ & Fan J (2019) Two‐stage metabolic remodelling in macrophages in response to lipopolysaccharide and interferon‐γ stimulation. Nat Metab 1, 731–742. 32259027 10.1038/s42255-019-0083-2 PMC7108803

[64] Mills EL, Kelly B, Logan A, Costa ASH, Varma M, Bryant CE, Tourlomousis P, Dabritz JHM, Gottlieb E, Latorre I*et al*. (2016) Succinate dehydrogenase supports metabolic repurposing of mitochondria to drive inflammatory macrophages. Cell 167, 457–470.e13. 27667687 10.1016/j.cell.2016.08.064 PMC5863951

[65] Booth AN, Taylor J, Wilson RH & Deeds F (1952) The inhibitory effects of itaconic acid in vitro and in vivo. J Biol Chem. 195, 697–702. 14946179

[66] Wu J‐Y, Huang T‐W, Hsieh Y‐T, Wang Y‐F, Yen C‐C, Lee G‐L, Yeh C‐C, Peng Y‐J, Kuo Y‐Y, Wen H‐T*et al*. (2020) Cancer‐derived succinate promotes macrophage polarization and cancer metastasis via succinate receptor. Mol Cell 77, 213–227.e5. 31735641 10.1016/j.molcel.2019.10.023

[67] Keiran N, Ceperuelo‐Mallafré V, Calvo E, Hernández‐Alvarez MI, Ejarque M, Núñez‐Roa C, Horrillo D, Maymó‐Masip E, Rodríguez MM, Fradera R*et al*. (2019) SUCNR1 controls an anti‐inflammatory program in macrophages to regulate the metabolic response to obesity. Nat Immunol 20, 581–592. 30962591 10.1038/s41590-019-0372-7

[68] Peruzzotti‐Jametti L, Bernstock JD, Vicario N, Costa ASH, Kwok CK, Leonardi T, Booty LM, Bicci I, Balzarotti B, Volpe G*et al*. (2018) Macrophage‐derived extracellular succinate licenses neural stem cells to suppress chronic neuroinflammation. Cell Stem Cell 22, 355–368.e13. 29478844 10.1016/j.stem.2018.01.020 PMC5842147

[69] Harber KJ, de Goede KE, Verberk SGS, Meinster E, de Vries HE, van Weeghel M, de Winther MPJ & Van den Bossche J (2020) Succinate is an inflammation‐induced immunoregulatory metabolite in macrophages. Metabolites 10, 372. 10.3390/metabo10090372 PMC7569821 32942769

[70] Littlewood‐Evans A, Sarret S, Apfel V, Loesle P, Dawson J, Zhang J, Muller A, Tigani B, Kneuer R, Patel S*et al*. (2016) GPR91 senses extracellular succinate released from inflammatory macrophages and exacerbates rheumatoid arthritis. J Exp Med 213, 1655–1662. 27481132 10.1084/jem.20160061 PMC4995082

[71] Trauelsen M, Rexen Ulven E, Hjorth SA, Brvar M, Monaco C, Frimurer TM & Schwartz TW (2017) Receptor structure‐based discovery of non‐metabolite agonists for the succinate receptor GPR91. Mol Metab 6, 1585–1596. 29157600 10.1016/j.molmet.2017.09.005 PMC5699910

[72] Newsholme P, Curi R, Gordon S & Newsholme EA (1986) Metabolism of glucose, glutamine, long‐chain fatty acids and ketone bodies by murine macrophages. Biochem J 239, 121–125. 3800971 10.1042/bj2390121 PMC1147248

[73] Newsholme P, Gordon S & Newsholme EA (1987) Rates of utilization and fates of glucose, glutamine, pyruvate, fatty acids and ketone bodies by mouse macrophages. Biochem J 242, 631–636. 3593269 10.1042/bj2420631 PMC1147758

[74] Newsholme P & Newsholme EA (1989) Rates of utilization of glucose, glutamine and oleate and formation of end‐products by mouse peritoneal macrophages in culture. Biochem J 261, 211–218. 2775207 10.1042/bj2610211 PMC1138802

[75] Drapier JC & Hibbs JB Jr (1986) Murine cytotoxic activated macrophages inhibit aconitase in tumor cells. Inhibition involves the iron‐sulfur prosthetic group and is reversible. J Clin Invest 78, 790–797. 3745439 10.1172/JCI112642 PMC423677

[76] Drapier JC & Hibbs JB Jr (1950) (1988) Differentiation of murine macrophages to express nonspecific cytotoxicity for tumor cells results in L‐arginine‐dependent inhibition of mitochondrial iron‐sulfur enzymes in the macrophage effector cells. J Immunol 140, 2829–2838. 2451695

[77] Brown GC & Cooper CE (1994) Nanomolar concentrations of nitric oxide reversibly inhibit synaptosomal respiration by competing with oxygen at cytochrome oxidase. FEBS Lett 356, 295–298. 7805858 10.1016/0014-5793(94)01290-3

[78] Cleeter MW, Cooper JM, Darley‐Usmar VM, Moncada S & Schapira AH (1994) Reversible inhibition of cytochrome c oxidase, the terminal enzyme of the mitochondrial respiratory chain, by nitric oxide. Implications for neurodegenerative diseases. FEBS Lett 345, 50–54. 8194600 10.1016/0014-5793(94)00424-2

[79] Clementi E, Brown GC, Feelisch M & Moncada S (1998) Persistent inhibition of cell respiration by nitric oxide: crucial role of *S*‐nitrosylation of mitochondrial complex I and protective action of glutathione. Proc Natl Acad Sci USA 95, 7631–7636. 9636201 10.1073/pnas.95.13.7631 PMC22706

[80] Jin Z, Wei W, Yang M, Du Y & Wan Y (2014) Mitochondrial complex I activity suppresses inflammation and enhances bone resorption by shifting macrophage‐osteoclast polarization. Cell Metab 20, 483–498. 25130399 10.1016/j.cmet.2014.07.011 PMC4156549

[81] Vogel RO, Janssen RJ, van den Brand MA, Dieteren CE, Verkaart S, Koopman WJ, Willems PH, Pluk W, van den Heuvel LP & Smeitink JA (2007) Cytosolic signaling protein Ecsit also localizes to mitochondria where it interacts with chaperone NDUFAF1 and functions in complex I assembly. Genes Dev 21, 615–624. 17344420 10.1101/gad.408407 PMC1820902

[82] West AP, Brodsky IE, Rahner C, Woo DK, Erdjument‐Bromage H, Tempst P, Walsh MC, Choi Y, Shadel GS & Ghosh S (2011) TLR signalling augments macrophage bactericidal activity through mitochondrial ROS. Nature 472, 476–480. 21525932 10.1038/nature09973 PMC3460538

[83] Kelly B, Tannahill GM, Murphy MP & O'Neill LA (2015) Metformin inhibits the production of reactive oxygen species from NADH: Ubiquinone oxidoreductase to limit induction of interleukin‐1β (IL‐1β) and boosts interleukin‐10 (IL‐10) in lipopolysaccharide (LPS)‐activated macrophages. J Biol Chem 290, 20348–20359. 26152715 10.1074/jbc.M115.662114 PMC4536441

[84] Mills EL, Kelly B, Logan A, Costa ASH, Varma M, Bryant CE, Tourlomousis P, Däbritz JHM, Gottlieb E, Latorre I*et al*. (2016) Succinate dehydrogenase supports metabolic repurposing of mitochondria to drive inflammatory macrophages. Cell 167, 457–470.e13. 27667687 10.1016/j.cell.2016.08.064 PMC5863951

[85] Garaude J, Acín‐Pérez R, Martínez‐Cano S, Enamorado M, Ugolini M, Nistal‐Villán E, Hervás‐Stubbs S, Pelegrín P, Sander LE, Enríquez JA & *et al*. (2016) Mitochondrial respiratory‐chain adaptations in macrophages contribute to antibacterial host defense. Nat Immunol 17, 1037–1045. 27348412 10.1038/ni.3509 PMC4994870

[86] Palmieri EM, Gonzalez‐Cotto M, Baseler WA, Davies LC, Ghesquière B, Maio N, Rice CM, Rouault TA, Cassel T, Higashi RM*et al*. (2020) Nitric oxide orchestrates metabolic rewiring in M1 macrophages by targeting aconitase 2 and pyruvate dehydrogenase. Nat Commun 11, 698. 32019928 10.1038/s41467-020-14433-7 PMC7000728

[87] Van den Bossche J, Baardman J, Otto NA, van der Velden S, Neele AE, van den Berg SM, Luque‐Martin R, Chen H‐J, Boshuizen MCS, Ahmed M*et al*. (2016) Mitochondrial dysfunction prevents repolarization of inflammatory macrophages. Cell Rep 17, 684–696. 27732846 10.1016/j.celrep.2016.09.008

[88] Kuhlencordt PJ, Chen J, Han F, Astern J & Huang PL (2001) Genetic deficiency of inducible nitric oxide synthase reduces atherosclerosis and lowers plasma lipid peroxides in apolipoprotein E‐knockout mice. Circulation 103, 3099–3104. 11425775 10.1161/01.cir.103.25.3099

[89] Niu XL, Yang X, Hoshiai K, Tanaka K, Sawamura S, Koga Y & Nakazawa H (2001) Inducible nitric oxide synthase deficiency does not affect the susceptibility of mice to atherosclerosis but increases collagen content in lesions. Circulation 103, 1115–1120. 11222475 10.1161/01.cir.103.8.1115

[90] Bailey JD, Diotallevi M, Nicol T, McNeill E, Shaw A, Chuaiphichai S, Hale A, Starr A, Nandi M, Stylianou E*et al*. (2019) Nitric oxide modulates metabolic remodeling in inflammatory macrophages through TCA cycle regulation and itaconate accumulation. Cell Rep 28, 218–230.e7. 31269442 10.1016/j.celrep.2019.06.018 PMC6616861

[91] McNeill E, Crabtree MJ, Sahgal N, Patel J, Chuaiphichai S, Iqbal AJ, Hale AB, Greaves DR & Channon KM (2015) Regulation of iNOS function and cellular redox state by macrophage Gch1 reveals specific requirements for tetrahydrobiopterin in NRF2 activation. Free Radic Biol Med 79, 206–216. 25451639 10.1016/j.freeradbiomed.2014.10.575 PMC4344222

[92] Mookerjee SA, Goncalves RLS, Gerencser AA, Nicholls DG & Brand MD (2015) The contributions of respiration and glycolysis to extracellular acid production. Biochim Biophys Acta – Bioenerget 1847, 171–181. 10.1016/j.bbabio.2014.10.005 25449966

[93] Everts B, Amiel E, van der Windt GJW, Freitas TC, Chott R, Yarasheski KE, Pearce EL & Pearce EJ (2012) Commitment to glycolysis sustains survival of NO‐producing inflammatory dendritic cells. Blood 120, 1422–1431. 22786879 10.1182/blood-2012-03-419747 PMC3423780

[94] Everts B, Amiel E, Huang SC, Smith AM, Chang CH, Lam WY, Redmann V, Freitas TC, Blagih J, van der Windt GJ*et al*. (2014) TLR‐driven early glycolytic reprogramming via the kinases TBK1‐IKKɛ supports the anabolic demands of dendritic cell activation. Nat Immunol 15, 323–332. 24562310 10.1038/ni.2833 PMC4358322

[95] Kennedy MC, Antholine WE & Beinert H (1997) An EPR investigation of the products of the reaction of cytosolic and mitochondrial aconitases with nitric oxide. J Biol Chem 272, 20340–20347. 9252338 10.1074/jbc.272.33.20340

[96] Yan LJ, Liu L & Forster MJ (2012) Reversible inactivation of dihydrolipoamide dehydrogenase by Angeli's salt. Sheng Wu Wu Li Hsueh Bao 28, 341–350. 23139597 PMC3490496

[97] Mishra BB, Rathinam VAK, Martens GW, Martinot AJ, Kornfeld H, Fitzgerald KA & Sassetti CM (2013) Nitric oxide controls the immunopathology of tuberculosis by inhibiting NLRP3 inflammasome–dependent processing of IL‐1β. Nat Immunol 14, 52–60. 23160153 10.1038/ni.2474 PMC3721324

[98] Schön T, Elias D, Moges F, Melese E, Tessema T, Stendahl O, Britton S & Sundqvist T (2003) Arginine as an adjuvant to chemotherapy improves clinical outcome in active tuberculosis. Europ Res J 21, 483–488. 10.1183/09031936.03.00090702 12662006

[99] Meiser J, Krämer L, Sapcariu SC, Battello N, Ghelfi J, D'Herouel AF, Skupin A & Hiller K (2016) Pro‐inflammatory macrophages sustain pyruvate oxidation through pyruvate dehydrogenase for the synthesis of itaconate and to enable cytokine expression. J Biol Chem 291, 3932–3946. 26679997 10.1074/jbc.M115.676817 PMC4759172

[100] Swain A, Bambouskova M, Kim H, Andhey PS, Duncan D, Auclair K, Chubukov V, Simons DM, Roddy TP, Stewart KM*et al*. (2020) Comparative evaluation of itaconate and its derivatives reveals divergent inflammasome and type I interferon regulation in macrophages. Nat Metab 2, 594–602. 32694786 10.1038/s42255-020-0210-0 PMC7378276

[101] Biswas SK & Lopez‐Collazo E (2009) Endotoxin tolerance: new mechanisms, molecules and clinical significance. Trends Immunol 30, 475–487. 19781994 10.1016/j.it.2009.07.009

[102] Zhang Q, Zhao K, Shen Q, Han Y, Gu Y, Li X, Zhao D, Liu Y, Wang C, Zhang X*et al*. (2015) Tet2 is required to resolve inflammation by recruiting Hdac2 to specifically repress IL‐6. Nature 525, 389–393. 26287468 10.1038/nature15252 PMC4697747

[103] Kleinnijenhuis J, Quintin J, Preijers F, Joosten LA, Ifrim DC, Saeed S, Jacobs C, van Loenhout J, de Jong D, Stunnenberg HG*et al*. (2012) Bacille Calmette‐Guerin induces NOD2‐dependent nonspecific protection from reinfection via epigenetic reprogramming of monocytes. Proc Natl Acad Sci USA 109, 17537–17542. 22988082 10.1073/pnas.1202870109 PMC3491454

[104] Quintin J, Saeed S, Martens JHA, Giamarellos‐Bourboulis EJ, Ifrim DC, Logie C, Jacobs L, Jansen T, Kullberg B‐J, Wijmenga C*et al*. (2012) Candida albicans infection affords protection against reinfection via functional reprogramming of monocytes. Cell Host Microbe 12, 223–232. 22901542 10.1016/j.chom.2012.06.006 PMC3864037

[105] Saeed S, Quintin J, Kerstens HHD, Rao NA, Aghajanirefah A, Matarese F, Cheng S‐C, Ratter J, Berentsen K, van der Ent MA*et al*. (2014) Epigenetic programming of monocyte‐to‐macrophage differentiation and trained innate immunity. Science 345, 1251086. 25258085 10.1126/science.1251086 PMC4242194

[106] Arts RJW, Novakovic B, ter Horst R, Carvalho A, Bekkering S, Lachmandas E, Rodrigues F, Silvestre R, Cheng S‐C, Wang S‐Y*et al*. (2016) Glutaminolysis and fumarate accumulation integrate immunometabolic and epigenetic programs in trained immunity. Cell Metab 24, 807–819. 27866838 10.1016/j.cmet.2016.10.008 PMC5742541

[107] Elliott P, Posma JM, Chan Q, Garcia‐Perez I, Wijeyesekera A, Bictash M, Ebbels TM, Ueshima H, Zhao L & Van Horn L (2015) Urinary metabolic signatures of human adiposity. Sci Transl Med. 7, 285ra62‐285ra62. 10.1126/scitranslmed.aaa5680 PMC6598200 25925681

[108] Wahl S, Yu Z, Kleber M, Singmann P, Holzapfel C, He Y, Mittelstrass K, Polonikov A, Prehn C & Römisch‐Margl W (2012) Childhood obesity is associated with changes in the serum metabolite profile. Obesity Facts 5, 660–670. 23108202 10.1159/000343204

[109] Cheng S, Rhee EP, Larson MG, Lewis GD, McCabe EL, Shen D, Palma MJ, Roberts LD, Dejam A, Souza AL*et al*. (2012) Metabolite profiling identifies pathways associated with metabolic risk in humans. Circulation 125, 2222–2231. 22496159 10.1161/CIRCULATIONAHA.111.067827 PMC3376658

[110] van Diepen JA, Robben JH, Hooiveld GJ, Carmone C, Alsady M, Boutens L, Bekkenkamp‐Grovenstein M, Hijmans A, Engelke UF & Wevers RA (2017) SUCNR1‐mediated chemotaxis of macrophages aggravates obesity‐induced inflammation and diabetes. Diabetologia 60, 1304–1313. 28382382 10.1007/s00125-017-4261-z PMC5487589

[111] Gmünder H, Eck HP, Benninghoff B, Roth S & Dröge W (1990) Macrophages regulate intracellular glutathione levels of lymphocytes. Evidence for an immunoregulatory role of cysteine. Cell Immunol 129, 32–46. 2364441 10.1016/0008-8749(90)90184-s

[112] Watanabe H & Bannai S (1987) Induction of cystine transport activity in mouse peritoneal macrophages. J Exp Med 165, 628–640. 2880923 10.1084/jem.165.3.628 PMC2188288

[113] Rouzer CA, Scott WA, Griffith OW, Hamill AL & Cohn ZA (1982) Glutathione metabolism in resting and phagocytizing peritoneal macrophages. J Biol Chem 257, 2002–2008. 6120172

[114] Thimmulappa RK, Lee H, Rangasamy T, Reddy SP, Yamamoto M, Kensler TW & Biswal S (2006) Nrf2 is a critical regulator of the innate immune response and survival during experimental sepsis. J Clin Investig 116, 984–995. 16585964 10.1172/JCI25790 PMC1421348

[115] Kobayashi EH, Suzuki T, Funayama R, Nagashima T, Hayashi M, Sekine H, Tanaka N, Moriguchi T, Motohashi H, Nakayama K*et al*. (2016) Nrf2 suppresses macrophage inflammatory response by blocking proinflammatory cytokine transcription. Nat Commun 7, 11624. 27211851 10.1038/ncomms11624 PMC4879264

[116] Wrotek S, Domagalski K, Jędrzejewski T, Dec E & Kozak W (2017) Buthionine sulfoximine, a glutathione depletor, attenuates endotoxic fever and reduces IL‐1β and IL‐6 level in rats. Cytokine 90, 31–37. 27764704 10.1016/j.cyto.2016.10.005

[117] Zhou R, Yazdi AS, Menu P & Tschopp J (2011) A role for mitochondria in NLRP3 inflammasome activation. Nature 469, 221–225. 21124315 10.1038/nature09663

[118] Wagner EJ & Carpenter PB (2012) Understanding the language of Lys36 methylation at histone H3. Nat Rev Mol Cell Biol 13, 115–126. 22266761 10.1038/nrm3274 PMC3969746

[119] Van Dyken SJ & Locksley RM (2013) Interleukin‐4‐ and interleukin‐13‐mediated alternatively activated macrophages: roles in homeostasis and disease. Annu Rev Immunol 31, 317–343. 23298208 10.1146/annurev-immunol-032712-095906 PMC3606684

[120] Gordon S (2003) Alternative activation of macrophages. Nat Rev Immunol 3, 23–35. 12511873 10.1038/nri978

[121] Stein M, Keshav S, Harris N & Gordon S (1992) Interleukin 4 potently enhances murine macrophage mannose receptor activity: a marker of alternative immunologic macrophage activation. J Exp Med 176, 287–292. 1613462 10.1084/jem.176.1.287 PMC2119288

[122] Gerrard TL, Dyer DR & Mostowski HS (1990) IL‐4 and granulocyte‐macrophage colony‐stimulating factor selectively increase HLA‐DR and HLA‐DP antigens but not HLA‐DQ antigens on human monocytes. J Immunol 144, 4670–4674. 2112573

[123] Donnelly RP, Fenton MJ, Finbloom DS & Gerrard TL (1990) Differential regulation of IL‐1 production in human monocytes by IFN‐gamma and IL‐4. J Immunol 145, 569–575. 2114443

[124] McBride WH, Economou JS, Nayersina R, Comora S & Essner R (1990) Influences of interleukins 2 and 4 on tumor necrosis factor production by murine mononuclear phagocytes. Cancer Res 50, 2949–2952. 2334896

[125] Standiford TJ, Strieter RM, Chensue SW, Westwick J, Kasahara K & Kunkel SL (1990) IL‐4 inhibits the expression of IL‐8 from stimulated human monocytes. J Immunol 145, 1435–1439. 2200823

[126] Mosmann TR & Coffman RL (1989) TH1 and TH2 cells: different patterns of lymphokine secretion lead to different functional properties. Annu Rev Immunol 7, 145–173. 2523712 10.1146/annurev.iy.07.040189.001045

[127] Mills CD, Kincaid K, Alt JM, Heilman MJ & Hill AM (2000) M‐1/M‐2 Macrophages and the Th1/Th2 Paradigm. J Immunol 164, 6166. 10843666 10.4049/jimmunol.164.12.6166

[128] McKenzie AN, Culpepper JA, de Waal Malefyt R, Brière F, Punnonen J, Aversa G, Sato A, Dang W, Cocks BG, Menon S*et al*. (1993) Interleukin 13, a T‐cell‐derived cytokine that regulates human monocyte and B‐cell function. Proc Natl Acad Sci U S A 90, 3735–3739. 8097324 10.1073/pnas.90.8.3735 PMC46376

[129] Mantovani A, Sica A, Sozzani S, Allavena P, Vecchi A & Locati M (2004) The chemokine system in diverse forms of macrophage activation and polarization. Trends Immunol 25, 677–686. 15530839 10.1016/j.it.2004.09.015

[130] Sica A & Mantovani A (2012) Macrophage plasticity and polarization: in vivo veritas. J Clin Invest 122, 787–795. 22378047 10.1172/JCI59643 PMC3287223

[131] Martinez FO, Helming L & Gordon S (2009) Alternative activation of macrophages: an immunologic functional perspective. Annu Rev Immunol 27, 451–483. 19105661 10.1146/annurev.immunol.021908.132532

[132] Shearer JD, Richards JR, Mills CD & Caldwell MD (1997) Differential regulation of macrophage arginine metabolism: a proposed role in wound healing. Am J Physiol‐Endocrinol Metab 272, E181–E190. 10.1152/ajpendo.1997.272.2.E181 9124321

[133] Mills CD, Shearer J, Evans R & Caldwell MD (1992) Macrophage arginine metabolism and the inhibition or stimulation of cancer. J Immunol 149, 2709. 1401910

[134] Qian BZ & Pollard JW (2010) Macrophage diversity enhances tumor progression and metastasis. Cell 141, 39–51. 20371344 10.1016/j.cell.2010.03.014 PMC4994190

[135] Muz B, de la Puente P, Azab F & Azab AK (2015) The role of hypoxia in cancer progression, angiogenesis, metastasis, and resistance to therapy. Hypoxia (Auckland, NZ) 3, 83–92. 10.2147/HP.S93413 PMC5045092 27774485

[136] Covarrubias AJ, Aksoylar HI, Yu J, Snyder NW, Worth AJ, Iyer SS, Wang J, Ben‐Sahra I, Byles V, Polynne‐Stapornkul T*et al*. (2016) Akt‐mTORC1 signaling regulates Acly to integrate metabolic input to control of macrophage activation. Elife 5, e11612. 26894960 10.7554/eLife.11612 PMC4769166

[137] Huang S‐C, Smith AM, Everts B, Colonna M, Pearce EL, Schilling JD & Pearce EJ (2016) Metabolic reprogramming mediated by the mTORC2‐IRF4 signaling axis is essential for macrophage alternative activation. Immunity 45, 817–830. 27760338 10.1016/j.immuni.2016.09.016 PMC5535820

[138] Van den Bossche J, O’Neill LA & Menon D (2017) Macrophage immunometabolism: Where are we (Going)? Trends Immunol 38, 395–406. 28396078 10.1016/j.it.2017.03.001

[139] Wang F, Zhang S, Vuckovic I, Jeon R, Lerman A, Folmes CD, Dzeja PP & Herrmann J (2018) Glycolytic stimulation is not a requirement for M2 macrophage differentiation. Cell Metab 28, 463–475.e4. 30184486 10.1016/j.cmet.2018.08.012 PMC6449248

[140] Sahin E, Haubenwallner S, Kuttke M, Kollmann I, Halfmann A, Dohnal AM, Chen L, Cheng P, Hoesel B, Einwallner E*et al*. (2014) Macrophage PTEN regulates expression and secretion of arginase I modulating innate and adaptive immune responses. J Immunol 193, 1717–1727. 10.4049/jimmunol.1302167 25015834 PMC4120896

[141] Brunner JS, Vulliard L, Hofmann M, Kieler M, Lercher A, Vogel A, Russier M, Brüggenthies JB, Kerndl M, Saferding V*et al*. (2020) Environmental arginine controls multinuclear giant cell metabolism and formation. Nat Commun 11, 431. 10.1038/s41467-020-14285-1 31969567 PMC6976629

[142] Schreiber T, Ehlers S, Heitmann L, Rausch A, Mages J, Murray PJ, Lang R & Hölscher C (2009) Autocrine IL‐10 induces hallmarks of alternative activation in macrophages and suppresses antituberculosis effector mechanisms without compromising T cell immunity. J Immunol 183, 1301. 19561100 10.4049/jimmunol.0803567 PMC2735238

[143] Pesce J, Ramalingam T, Mentink‐Kane M, Wilson M, El Kasmi K, Smith A, Thompson R, Cheever A, Murray P & Wynn T (2009) Arginase‐1‐expressing macrophages suppress Th2 cytokine‐driven inflammation and fibrosis. PLoS Pathog 5, e1000371. 19360123 10.1371/journal.ppat.1000371 PMC2660425

[144] Tavares J, Ouaissi A, Lin PKT, Tomás A & Cordeiro‐da‐Silva A (2005) Differential effects of polyamine derivative compounds against Leishmania infantum promastigotes and axenic amastigotes. Int J Parasitol 35, 637–646. 15862577 10.1016/j.ijpara.2005.01.008

[145] Esser‐von Bieren J, Mosconi I, Guiet R, Piersgilli A, Volpe B, Chen F, Gause WC, Seitz A, Verbeek JS & Harris NL (2013) Antibodies trap tissue migrating helminth larvae and prevent tissue damage by driving IL‐4Rα‐independent alternative differentiation of macrophages. PLoS Pathog 9, e1003771. 24244174 10.1371/journal.ppat.1003771 PMC3828184

[146] Pegg AE (2016) Functions of polyamines in mammals. J Biol Chem 291, 14904–14912. 27268251 10.1074/jbc.R116.731661 PMC4946908

[147] Miller‐Fleming L, Olin‐Sandoval V, Campbell K & Ralser M (2015) Remaining mysteries of molecular biology: The role of polyamines in the cell. J Mol Biol 427, 3389–3406. 26156863 10.1016/j.jmb.2015.06.020

[148] Van den Bossche J, Lamers WH, Koehler ES, Geuns JMC, Alhonen L, Uimari A, Pirnes‐Karhu S, Van Overmeire E, Morias Y, Brys L*et al*. (2012) Pivotal advance: Arginase‐1‐independent polyamine production stimulates the expression of IL‐4‐induced alternatively activated macrophage markers while inhibiting LPS‐induced expression of inflammatory genes. J Leukoc Biol 91, 685–699. 22416259 10.1189/jlb.0911453

[149] Puleston DJ, Buck MD, Klein Geltink RI, Kyle RL, Caputa G, O’Sullivan D, Cameron AM, Castoldi A, Musa Y, Kabat AM*et al*. (2019) Polyamines and eIF5A hypusination modulate mitochondrial respiration and macrophage activation. Cell Metab 30, 352–363.e8. 31130465 10.1016/j.cmet.2019.05.003 PMC6688828

[150] Yurdagul A Jr, Subramanian M, Wang X, Crown SB, Ilkayeva OR, Darville L, Kolluru GK, Rymond CC, Gerlach BD, Zheng Z*et al*. (2020) Macrophage metabolism of apoptotic cell‐derived arginine promotes continual efferocytosis and resolution of injury. Cell Metab 31, 518–533.e10. 32004476 10.1016/j.cmet.2020.01.001 PMC7173557

[151] Thomas AC & Mattila JT (2014) "Of mice and men": arginine metabolism in macrophages. Front Immunol 5, 479. 25339954 10.3389/fimmu.2014.00479 PMC4188127

[152] Sivangala Thandi R, Radhakrishnan RK, Tripathi D, Paidipally P, Azad AK, Schlesinger LS, Samten B, Mulik S & Vankayalapati R (2020) Ornithine‐A urea cycle metabolite enhances autophagy and controls Mycobacterium tuberculosis infection. Nat Commun 11, 3535. 32669568 10.1038/s41467-020-17310-5 PMC7363810

[153] Gutierrez MG, Master SS, Singh SB, Taylor GA, Colombo MI & Deretic V (2004) Autophagy is a defense mechanism inhibiting BCG and Mycobacterium tuberculosis survival in infected macrophages. Cell 119, 753–766. 15607973 10.1016/j.cell.2004.11.038

[154] Ishii M, Wen H, Corsa CA, Liu T, Coelho AL, Allen RM, Carson WFT, Cavassani KA, Li X, Lukacs NW, Hogaboam CM, Dou Y & Kunkel SL (2009) Epigenetic regulation of the alternatively activated macrophage phenotype. Blood 114, 3244–3254. 19567879 10.1182/blood-2009-04-217620 PMC2759649

[155] Ren W, Xia Y, Chen S, Wu G, Bazer FW, Zhou B, Tan B, Zhu G, Deng J & Yin Y (2019) Glutamine metabolism in macrophages: a novel target for obesity/type 2 diabetes. Adv Nutr. 10, 321–330. 30753258 10.1093/advances/nmy084 PMC6416106

[156] Wilmore DW (2543S) The effect of glutamine supplementation in patients following elective surgery and accidental injury. J Nut 131, 2543S–2549S. 10.1093/jn/131.9.2543S 11533310

[157] Palmieri EM, Menga A, Martin‐Perez R, Quinto A, Riera‐Domingo C, De Tullio G, Hooper DC, Lamers WH, Ghesquiere B, McVicar DW*et al*. (2017) Pharmacologic or genetic targeting of glutamine synthetase skews macrophages toward an M1‐like phenotype and inhibits tumor metastasis. Cell Rep 20, 1654–1666. 28813676 10.1016/j.celrep.2017.07.054 PMC5575233

[158] Huang JT, Welch JS, Ricote M, Binder CJ, Willson TM, Kelly C, Witztum JL, Funk CD, Conrad D & Glass CK (1999) Interleukin‐4‐dependent production of PPAR‐γ ligands in macrophages by 12/15‐lipoxygenase. Nature 400, 378–382. 10432118 10.1038/22572

[159] Nelson VL, Nguyen HCB, Garcia‐Canaveras JC, Briggs ER, Ho WY, DiSpirito JR, Marinis JM, Hill DA & Lazar MA (2018) PPARgamma is a nexus controlling alternative activation of macrophages via glutamine metabolism. Genes Dev 32, 1035–1044. 30006480 10.1101/gad.312355.118 PMC6075146

[160] Davies LC, Rice CM, Palmieri EM, Taylor PR, Kuhns DB & McVicar DW (2017) Peritoneal tissue‐resident macrophages are metabolically poised to engage microbes using tissue‐niche fuels. Nat Commun 8, 2074. 29234000 10.1038/s41467-017-02092-0 PMC5727035

[161] Weiss JM, Davies LC, Karwan M, Ileva L, Ozaki MK, Cheng RY, Ridnour LA, Annunziata CM, Wink DA & McVicar DW (2018) Itaconic acid mediates crosstalk between macrophage metabolism and peritoneal tumors. J Clin Invest 128, 3794–3805. 29920191 10.1172/JCI99169 PMC6118601

[162] Oh MH, Collins SL, Sun IH, Tam AJ, Patel CH, Arwood ML, Chan‐Li Y, Powell JD & Horton MR (2017) mTORC2 signaling selectively regulates the generation and function of tissue‐resident peritoneal macrophages. Cell Rep 20, 2439–2454. 28877476 10.1016/j.celrep.2017.08.046 PMC5659290

[163] Krausgruber T, Fortelny N, Fife‐Gernedl V, Senekowitsch M, Schuster LC, Lercher A, Nemc A, Schmidl C, Rendeiro AF, Bergthaler A*et al*. (2020) Structural cells are key regulators of organ‐specific immune responses. Nature 583, 296–302. 32612232 10.1038/s41586-020-2424-4 PMC7610345

[164] Murray PJ & Wynn TA (2011) Obstacles and opportunities for understanding macrophage polarization. J Leukoc Biol 89, 557–563. 21248152 10.1189/jlb.0710409 PMC3058818

[165] Murray PJ (2017) Macrophage polarization. Annu Rev Physiol 79, 541–566. 27813830 10.1146/annurev-physiol-022516-034339

[166] Murray PJ (2020) On macrophage diversity and inflammatory metabolic timers. Nat Rev Immunol 20, 89–90. 31804612 10.1038/s41577-019-0260-2

